# Detection of Turbulent Combustion Mechanism in NH_3_/H_2_ Stratified Premixtures Using Chemical Explosive Mode Analysis

**DOI:** 10.1021/acsomega.5c05835

**Published:** 2025-10-22

**Authors:** Yinhu Kang, Jiuyi Zhang, Wenxuan Zhou, Haoran Wang, Xiaomei Huang, Xiaofeng Lu

**Affiliations:** † Key Laboratory of Low-Grade Energy Utilization Technologies and Systems, (Chongqing University), Ministry of Education of China, Chongqing 400044, China; ‡ School of Civil Engineering, 47913 Chongqing University, Chongqing 400044, China

## Abstract

The turbulent combustion process of NH_3_/H_2_/air stratified premixtures is simulated by DNS, which shows the existence of diverse local modes and structures in the domain. The chemical explosive mode analysis (CEMA) diagnostic tool, which has systematic and rigorous diagnostic capabilities, is employed to clarify the fundamental physical mechanics underlying the local modes, structures, and events. It is found that a small temperature fluctuation corresponds to the spontaneous ignition mode, and an intermediate or large temperature fluctuation corresponds to the deflagration propagation mode. The dependence of the combustion mode on composition stratification is unimportant in the simulated cases. Compared to the spontaneous ignition mode, the combustion/flame interaction in the deflagration mode is inhibited due to the mitigation of thermal dilation, thereby leaving much finer structures in the domain. The “diffusion-assisted spontaneous ignition mode”, where diffusion ahead of the reaction front promotes spontaneous ignition and flame propagation, is a unique behavior in stratified NH_3_/H_2_/air combustion at elevated temperature fluctuation conditions. It is characterized by distinct structures that differ from the traditional autoignition mode and laminar deflagration. The preferential diffusion of H_2_/H radicals from the tailing-rich front to the leading leaner zone enhances the upstream ignition reactions, which is the underlying physics for the unique “diffusion-assisted spontaneous ignition mode” surviving in stratified NH_3_/H_2_ combustion. The local modes ahead of the ignition front are primarily determined by the flame curvature. More specifically, for ignition fronts with negative curvature, the concave front wrinkling to the unburnt side promotes spontaneous ignition; while for those with positive curvature, the tangential diffusion with a negative contribution to ignition appears just ahead of the propagating fronts, thereby leading to the presence of an extinction mode. For the stratified NH_3_/H_2_ mixture combustion, the preferential diffusion associated with H_2_/H radicals and tangential diffusion with a positive curvature are the most effective in promoting the local reaction rates ahead of the ignition front.

## Graphical abstract

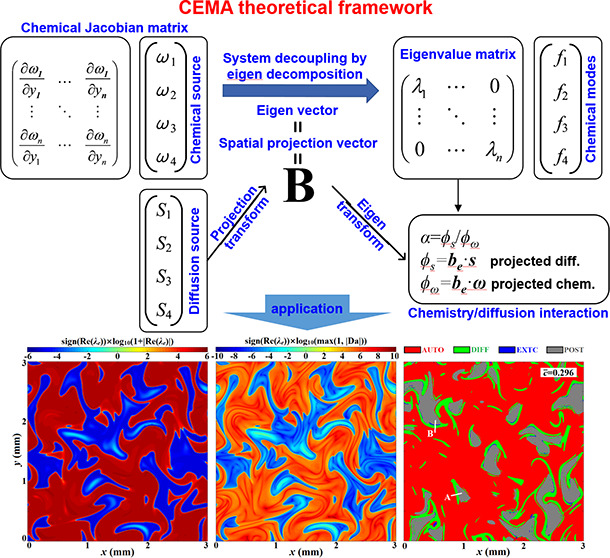

## 1 Introduction

Due to the impact of global warming, ammonia, a quintessential carbon-free fuel, has attracted growing attention in recent years. Ammonia fuel offers several advantages over traditional fuels, including a wide range of source bases, ease of storage and transportation, and high combustion energy density, making it a promising fuel candidate for gas engines and electric power generation. However, the widespread adoption of ammonia fuel is hindered by its unique combustion characteristics. Compared to traditional fossil fuels, ammonia faces challenges such as difficulty in achieving reliable ignition, lower laminar flame speed, poor flame stability, narrow flammability range, and significantly higher emissions of NOx and hydrogen. These issues are often mitigated by the blending of small quantities of highly reactive fuels (such as hydrogen, methane, or dimethyl ether) or by employing advanced combustion assistance techniques (such as porous media burners or plasma-assisted combustion).^1,2^ As another ideal carbon-free fuel candidate, hydrogen (H_2_) can be produced by the thermal dissociation of ammonia. Consequently, utilizing H_2_ as an additive to enhance ammonia combustion is inexpensive to implement, and this research holds significant fundamental and practical value. The blending of NH_3_ and H_2_ for combustion not only ensures zero carbon emissions but also yields much better flammability and stability behaviors than ammonia, thereby providing an effective strategy for the efficient and reliable utilization of ammonia. With respect to terminal gas equipment, the homogeneous charge compression ignition (HCCI) engine is considered a promising innovation for next-generation internal combustion engines. HCCI engines combine the advantages of premixed combustion of SI engines with the autoignition mechanics of CI engines, thereby featuring efficient and clean performance. However, the critical challenges of HCCI engines are how to mitigate the pressure rise rate (PRR) within the cylinder and simultaneously achieve precise control of the ignition timing, particularly under high-load conditions. To address this challenge, the stratified charge ignition technique, which can be achieved through various injection strategies, has shown prominent effectiveness in overcoming these issues. Furthermore, it is well accepted that ammonia is much more suitable for HCCI engine applications since the PRR is significantly retarded in ammonia combustion. In the meantime, the addition of H_2_ to NH_3_ will inevitably change the competing chemical pathways and differential diffusion in ammonia combustion, and thus the global engine performance. Hence, the combustion mechanism of NH_3_/H_2_ stratified mixtures under HCCI engine conditions is fundamentally and practically meaningful.

Lipatnikov et al.^3^ reviewed the mechanism of turbulent stratified combustion. Most previous studies have focused on the stratification of components with different equivalence ratios,^4–10^ elucidating the mechanism by which the instantaneous flame velocity in the stratified mixture deviates from that in the homogeneous mixture, known as the “antisupport” effect. This effect is attributed to changes in the heat diffusion flux from the product zone to the reaction zone. In the study by Tomidokoro et al.,^11^ numerical simulations and dynamic analyses were conducted to investigate the propagation mechanics of NH_3_/air laminar stratified premixed flames. The results indicated that at varying local equivalence ratio conditions, the flame propagation velocity of the stratified mixture exceeded that of the corresponding homogeneous mixture, regardless of whether the mixture was fuel-rich or lean. Moreover, the flame propagation velocity was observed to decrease with a reduction in the equivalence ratio, which can be attributed to a change in the H_2_ content within the product gas. Additionally, nitric oxide (NO) reduction reactions were identified in the stratified reacting flames, resulting in lower NO emissions as compared to the homogeneously premixed flame. Researchers have also focused on the implementation of two-chamber combustion systems with varying fuel reactivity stratifications, wherein the precombustion chamber utilizes a thermal jet to promote the ignition and combustion of the lean mixture in the main chamber, thereby enhancing the overall thermal and fuel efficiency. Chi et al.^12^ investigated the turbulent stratified combustion mechanism of ammonia in the main chamber, which was assisted by a prechamber hydrogen turbulent jet ignition (TJI), through a three-dimensional direct numerical simulation (DNS). They employed chemical explosive mode analysis (CEMA) to examine the flame thickness and propagation velocity of the turbulent premixed flame in the ammonia-hydrogen prechamber, as well as the structure of the turbulent stratified flame in the main chamber. The results indicated that the thickness of the stratified flame increased as it propagated into the NH_3_/air mixture. The overall structure of the flame front can be accurately approximated by the pyrolysis rate of the stratified flame, allowing for the identification of ignition, propagation, and extinction behaviors during combustion.

Yang et al.^13^ carried out simulations to explore the combustion characteristics of ammonia and ammonia–hydrogen blends in the spatially homogeneous reactor as well as the turbulent mixing layer at elevated pressures. The findings revealed the presence of two peaks in the heat release rate (HRR) during the latter spontaneous combustion period, as the flame propagation shifted toward the fuel-rich side in both laminar and turbulent flows. One peak is located at the isocontour of the high-temperature ignition mixture, which is primarily associated with reactions involving species such as hydroxyl radicals (OH), while the other peak corresponds to temperature increases that facilitate further flame propagation toward the fuel-rich fresh mixtures. The effects of elevated pressure and hydrogenation reactions on the position of spontaneous combustion were found to be consistent. Liang et al.^14^ conducted a pioneering study of the planar freely propagating premixed NH_3_ flames using reactivity stratification with H_2_ addition. It was shown that even when the entire flame structure resides on the low-reactivity ammonia side, flame propagation can be self-sustained, originating from the high-reactivity hydrogen. The hydrogen molecule that is responsible for reactivity stratification can arise from the thermal dissociation of ammonia. Key reactions contributing to this in situ generation of hydrogen include NH_*x*_ + H, as well as those involving HNO and N_2_H_2_. Furthermore, the elevated temperature of hydrogen combustion acts as a thermal support behind the ammonia flame. Yoo et al.^15^ performed DNS to examine the influence of thermal stratification on the ignition of a thin, inhomogeneous *n*-heptane/air premixture. It was indicated that both the mean and root-mean-square (RMS) of thermal stratification significantly affected the ignition delay time, depending on the ratio of the turbulence time scale to ignition delay time. A higher degree of thermal stratification tends to promote the deflagrative propagation mode rather than the spontaneous ignition mode.

In a study of turbulent stratified combustion under HCCI engine conditions, Pochet et al.^16^ evaluated the performance metrics of an ammonia–hydrogen hybrid HCCI engine, including combustion efficiency, indicated thermal efficiency, and output power at an effective compression ratio of 22. Their findings offer valuable insights for the design and optimization of ammonia–hydrogen HCCI engines. Kang et al.^17^ studied the combustion mechanism of ammonia and ammonia–hydrogen mixtures in the intensely turbulent jet mixing layer under spontaneous ignition conditions by high-order accuracy DNS. The fuel ignition dynamics, dominant combustion modes, and fundamental regimes governing the chemistry/diffusion interaction inside the turbulent jet mixing layer were elucidated by the transport budget analysis and conditional statistics. Tian et al.^18^ undertook an in-depth analysis of NH_3_/air combustion in the HCCI condition using a two-dimensional DNS. It was suggested that both turbulence and thermal stratification considerably favored the ignition process, with the ignition delay time increasing as the hydrogen addition ratio increased. In scenarios with elevated hydrogen contents, the HRR in regions with positive curvature was remarkably augmented due to the preferential diffusion of hydrogen. Notably, the global HRR level elevated at low-temperature fluctuation conditions, while under large temperature fluctuations, heat production tended to concentrate within a narrower region. By the decomposition of the flame speed and transport budget analysis, it was discerned that the combustion mode transitioned from spontaneous ignition at small temperature fluctuations to deflagration propagation at large temperature fluctuations. Bansal et al.^19^ examined the interaction mechanism between turbulent mixing and spontaneous reactions in dimethyl ether (DME)/air stratified premixes in an HCCI engine environment using DNS simulations in a constant-volume domain with an inert mass source term introduced into the continuity equation to mimic the in-cylinder pressure change resulting from piston movement in a practical scenario. Various diagnostic methods were employed to identify the diverse combustion modes occurring inside the domain, including spontaneous ignition, autoignition front propagation, and gas mixing, and to quantify the contributions of each mode to the overall HRR.

Despite the previous advancements in stratified NH_3_/H_2_ mixture combustion, the understanding nowadays of the physical mechanics underlying the diverse combustion modes under HCCI engine conditions, as well as the fundamental roles of diffusive transport and flame/flow interaction, is still quite insufficient. This paper investigates the combustion mechanism of NH_3_/H_2_ turbulent stratified premixtures at elevated pressures and initial temperatures using two-dimensional DNS with detailed fuel chemistry and transport models. To make a rigorous detection of the local combustion modes and quantify contributions of diffusive subprocesses to the local modes, we utilize the CEMA method to carry out systematic numerical diagnostics. The findings of this review are expected to provide a robust foundation for the optimization and control of NH_3_/H_2_-fired turbulent combustion systems, thereby advancing the technology of HCCI engines.

## 2 DNS Parameters Using Numerical Methods

DNS directly solves the Navier–Stokes equations along with the energy and species transport equations in the low-Mach formulation at the smallest spatial and time scales, without any simplifying assumptions or closure model; therefore, it can provide the most detailed information on turbulent combustion. In this study, DNS simulations were carried out using our in-house code written in Fortran and MPI for massively parallel computation. Details regarding the equations and methods can be found in our previous articles,^17,20^ and here we just give a brief description. The CHEMKIN^21^ and TRANSPORT codebases^22^ are integrated into the DNS code to calculate the chemical kinetics parameters and thermodynamic and transport properties of species. The multicomponent diffusion model was used while neglecting the pressure gradient dependence of the species diffusion velocity. Additionally, the viscous heating term and gravitational forces were neglected, while the optically thin radiation model^23^ was activated to account for thermal radiative loss. The radiating point source in the energy equation is estimated as *q*_r_ = 4σ∑_*i*=1_^4^(*p*_*i*_ *a*_*pi*_)×*T* ^4^ – *T*_b_^4^, where σ = 5.67 × 10^–8^ W/m^2^K^4^ is the Steffan–Boltzmann constant; *p*_*i*_ and *a*_*pi*_ are the partial pressure and Planck mean absorption coefficient of species *i*, respectively; *T* is the local flame temperature, and *T*_b_ is the ambient temperature, accounting for the contributions of four radiating species, including CO_2_, H_2_O, CH_4_, and CO. The spatial discretization of the governing equations is performed using an eighth-order central difference scheme,^24^ while the temporal integration is advanced using a fourth-order explicit Runge–Kutta method.^25^ An explicit numerical filtering scheme with tenth-order accuracy^24^ is employed to remove the nonphysical, high-frequency spurious numerical oscillations that may accumulate during the iteration process.

Table 1 presents the DNS initiation parameters for the generation of turbulent stratified mixtures. The fuel composition is assumed as nitrogen-diluted gas {30%[(1 – ω_H_2__)­NH_3_/ω_H_2__H_2_] + 70%N_2_}, where ω_H_2__ denotes the hydrogen molar ratio in the NH_3_/H_2_ mixture, and the oxidizer is exactly air (79%N_2_/21%O_2_). To reduce the overall computational cost, it was assumed that the in-cylinder injections were completed, and the initial field mimicked the stratifications of the temperature and stoichiometry resulting from the direct injection. An isotropic decaying turbulence field with a fluctuating velocity of *u*′ = 200 cm/s and a turbulence integral length scale of *l*_t_ = 0.4 mm was generated and then superimposed onto the laminar mean flow. It is noteworthy that a pseudospectral code based on Rogallo’s method,^26–28^ with the Passot–Pouquet energy spectrum model^29^ was used to generate the 2D turbulence, which can guarantee not only divergence-free (mass conservation) but also homogeneous isotropy of the turbulence. Additionally, temperature (*T*′) and equivalence ratio (ϕ′) fluctuations in the turbulence field were generated in a way similar to the turbulence energy spectrum model, using an identical integral length scale but distinct root-mean-square (RMS) values of the fluctuations. The resultant *T*′ and ϕ′ fluctuating fields were then superimposed onto the corresponding mean fields, i.e., ϕ_0_ and *T*_0_, respectively. Here, the initial temperature mean *T*_0_ = 1080 K and pressure *p*_0_ = 40 atm were kept constant for the current DNS simulations. Besides, recognizing that the liquid fuel injection into the cylinder absorbs latent heat when evaporating, the stratifications of temperature and equivalence ratio were assumed to be negatively correlated, i.e., *T*′ and ϕ′ always had a constant phase difference π in the Passot–Pouquet model formulation. For instance, Figure 1a,b show the initial profiles of temperature and vorticity for case 1, and Figure 1c shows that *T* and ϕ in the initial solution are absolutely negatively correlated.

**Figure 1:**
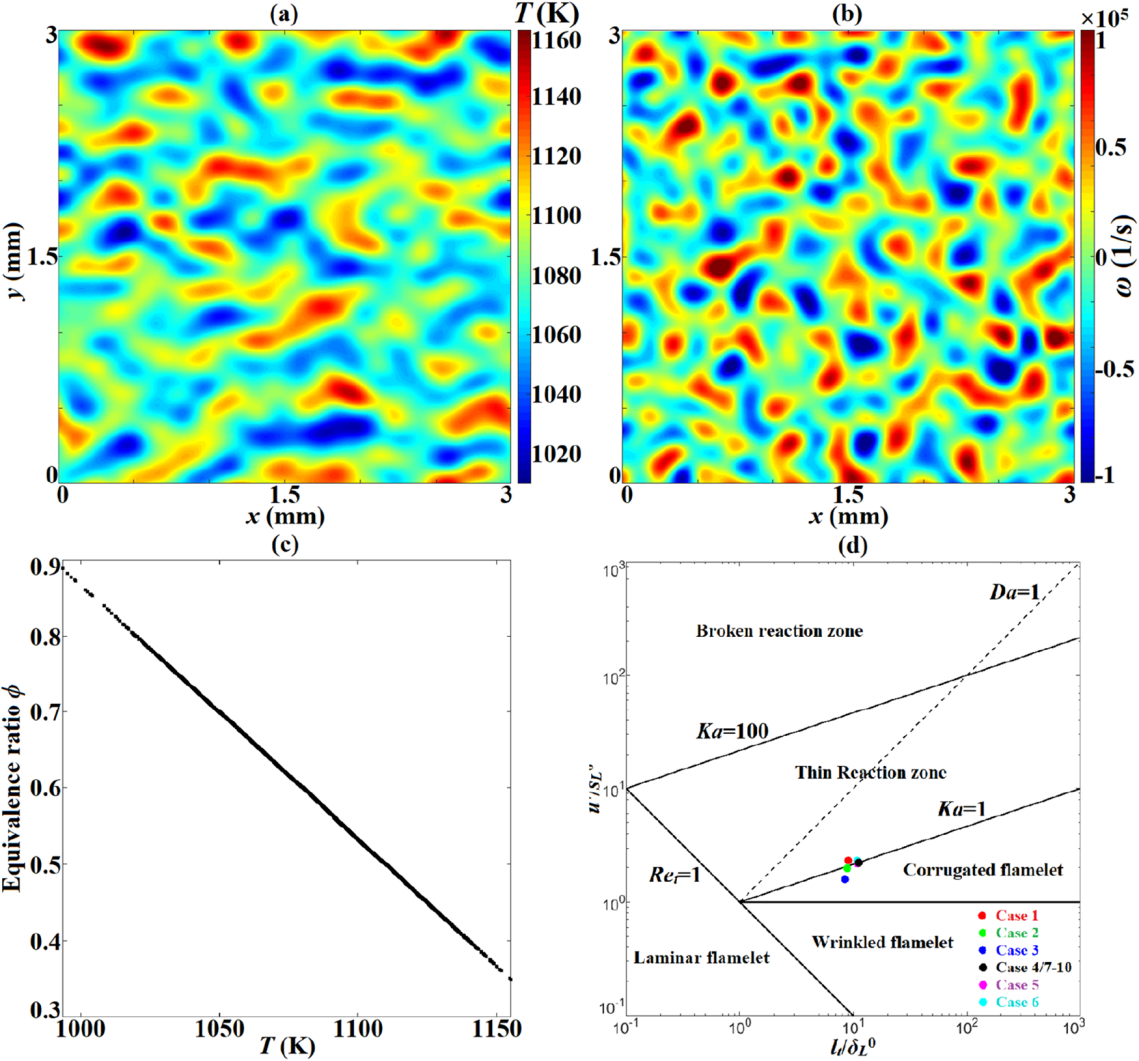
Initial profiles of temperature (a), vorticity (b), and *T*–ϕ correlation (c) in case 1. Location of cases 1–10 in the regime of the Borghi and Peters diagram (d).

**Table 1:** Initiation Parameters for the Turbulent Stratified Combustion in the Current DNS Simulations^a^

case	ω_H_2__	*T*_0_ (K)	*T*′ (K)	*T*′/⟨*T*⟩	ϕ_0_	ϕ′	*u*′ (cm/s)	*l*_t_ (mm)	*Re*_t_	η (μm)	*Da*_t_
1	0.1	1080	30	0.0278	0.6	0.1	200	0.4	223.0	6.93	0.25
2	0.2	1080	30	0.0278	0.6	0.1	200	0.4	221.2	6.97	0.32
3	0.3	1080	30	0.0278	0.6	0.1	200	0.4	219.2	7.02	0.38
4	0.1	1080	30	0.0278	0.8	0.1	200	0.4	221.5	6.97	0.25
5	0.1	1080	30	0.0278	1.0	0.1	200	0.4	220.3	7.00	0.25
6	0.1	1080	30	0.0278	1.2	0.1	200	0.4	219.3	7.02	0.25
7	0.1	1080	30	0.0278	0.8	0.2	200	0.4	221.5	6.97	0.25
8	0.1	1080	30	0.0278	0.8	0.3	200	0.4	221.5	6.97	0.25
9	0.1	1080	60	0.0556	0.8	0.1	200	0.4	221.5	6.97	0.25
10	0.1	1080	100	0.0926	0.8	0.1	200	0.4	221.5	6.97	0.25

a ⟨*T*⟩: volumetric mean of temperature; *u*′: RMS of the turbulent fluctuating velocity; *l*_t_: turbulence integral length scale; *Re*_t_: turbulent Reynolds number, *Re*_t_ = *u*′·*l*_t_ /*ν*, where *ν* is the fluid viscosity; η: Kolmogorov length scale, η = *l*_t_/*Re*_t_^0.75^; *Da*_t_: Turbulence Damköhler number, *Da*_t_ = (*l*_*t*_/*u′*)/τ_ig_^0D^, where τ_ig_^0D^ is the ignition delay time of the homogeneous mixture.

As listed in Table 1, cases 1/2/3 with varying ω_H_2__ values are designed to minimize the impact of hydrogen addition. Similarly, cases 1/4/5/6 are designed for clarifying the impact of the equivalence ratio mean (ϕ_0_), cases 4/7/8 are designed for equivalence ratio fluctuation (ϕ′), and cases 4/9/10 are designed for temperature fluctuation (*T*′). It is worth noting that the temperature fluctuations in HCCI engines prior to ignition are approximately 15–20 K,^15^ which are significantly lower than *T*′ values listed in Table 1 (30/60/100 K). However, employing direct or delayed injection strategies in HCCI engines can induce elevated temperature fluctuations, thereby enabling a broader temperature fluctuation range. The normalized temperature fluctuation (*T*′/⟨*T*⟩, where ⟨*T*⟩ is the volumetric mean of the temperature) is also shown in Table 1. Figure 1d depicts the location of each case in Borghi and Peters’ diagram,^30^ which shows the residence of cases 1–10 on the isoline *Ka* = 1. Here, *Ka* represents the ratio of the flamelet time scale to the Kolmogorov time scale; thus, *Ka* = 1 is regarded as the transition from the corrugated flame regime to the thin reaction zone regime. Hence, the role of turbulence–flame interactions was limited, and the dependence of stratified combustion on ω_H_2__, ϕ_0_, ϕ′, and *T*′ was augmented.

The 3D mesh can well capture the natural turbulence cascade interplay, flame stretching, and autoignition tendencies in the HCCI engine. However, considering the high cost of 3D DNS for turbulent combustion with detailed fuel chemistry, in the present study, the DNS was performed using a 2D mesh, which is not only cost-affordable but also can yield qualitatively the same results as the 3D DNS.^13,15,17^ The two-dimensional box domain (3 × 3 mm^2^) with periodic boundaries was discretized by a uniform mesh, with the grid size Δ*x* = Δ*y* = 2 μm, which is much smaller than the Kolmogorov length scale (η ≈ 7 μm), ensuring at least 50 grid points across the flame thickness (δ_f_). The laminar flame sheet thickness is estimated by δ_f_ = (*T*_ad_ – *T*_u_)/(∂*T*/∂*x*)_max_, where *T*_ad_ and *T*_u_ are the product temperature and unburned temperature, respectively. As a result, the resulting mesh is sufficiently fine to resolve the smallest length scale in the turbulence spectrum. A constant time step size Δ*t* = 2 ns, which falls fairly well within the Courant–Friedrichs–Lewy (CFL) conditions (i.e., *c*Δ*t*/Δ*x* < 1, where *c* is the sound speed), was used to ensure numerical convergence.

The ammonia chemical mechanism was developed by Konnov et al.,^31^ which contains the hydrogen submechanism and consists of 31 species and 241 elementary reactions, and was used for DNS simulations. To verify its accuracy, the homogeneous ignition delay times, predicted by Konnov’s mechanism,^31^ Han’s mechanism,^32^ Liao’s mechanism,^33^ Liu’s mechanism,^34^ Shrestha’s mechanism,^35^ and Thomas’ mechanism,^36^ are compared with the experimental data reported by Chen et al.^37^ for the NH_3_/H_2_ blended fuels, as shown in Figure 2. The results obtained using Konnov’s mechanism agree fairly well with those of the other mechanisms as well as the measurement data.

**Figure 2:**
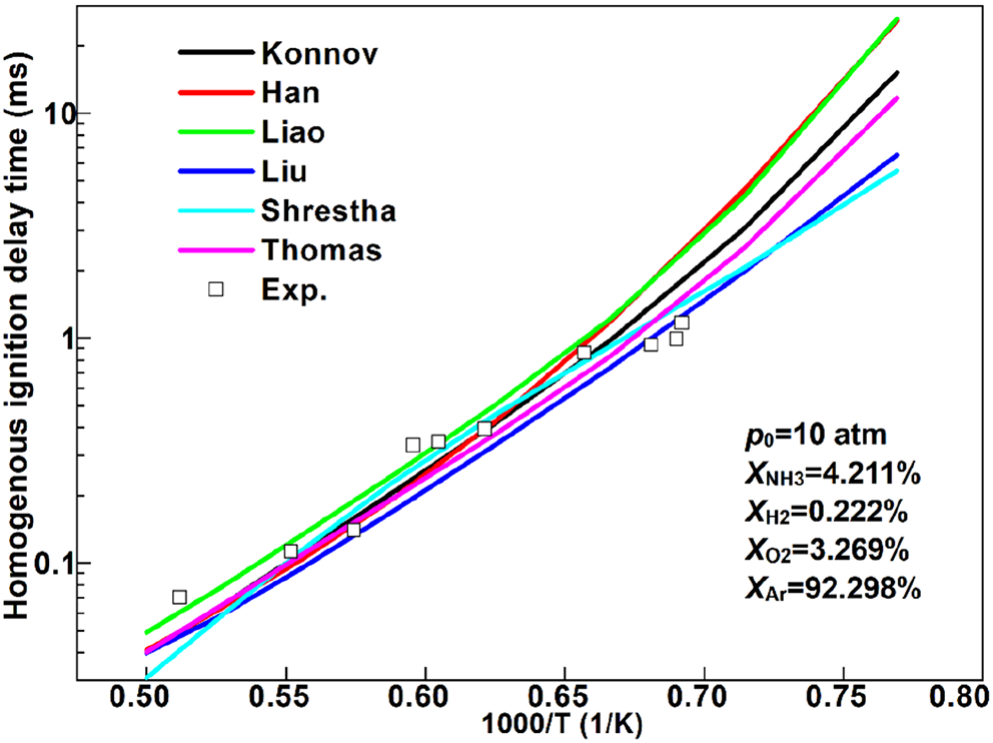
Predicted homogeneous ignition delay times using various ammonia chemical mechanisms (lines) as well as the corresponding experimental data (symbols) with respect to the reciprocal of the initial temperature.

## 3 Chemical Explosive Mode Analysis (CEMA)

CEMA is a systematic diagnostic tool for characterizing ignition, extinction, and flame dynamics.^38^ It effectively characterizes various critical events such as ignition, extinction, combustion stability, and propagation front, elucidating the dominant physical variables and reactions with the aid of appropriate criteria. The CEMA methodology computes the eigenvalues via the eigen-decomposition of the chemical Jacobian matrix (***J***_**ω**_) of the governing equations, which encapsulates the chemical kinetics of the local mixture. The chemical explosive mode (CEM) is defined as the reaction mode with an eigenvalue with a positive real part (i.e., Re­(λ_e_)>0), with the corresponding right and left eigenvectors denoted as **a_e_** and **b_e_**, respectively. The eigenvalue (λ_e_) with the corresponding eigenvectors (**a_e_**, **b_e_**) associated with the CEM mode satisfies the criteria of λ_e_ = **b_e_** · ***J***_**ω**_·**a_e_**. The CEM mode quantifies the spontaneous explosion tendency of mixtures in an adiabatically closed environment. In cases where multiple CEMs are present, the one with the largest real part magnitude is assumed to govern the overall explosive rate of the ignition process. However, in the postignition phase, as the combustion approaches the equilibrium, the CEM mode vanishes, and the governing mode satisfies Re­(λ_e_) < 0. In the framework of CEMA, the explosion index (EI) and participation index (PI) are valued in the span [0,1], as defined in eqs 1 and 2,^39,40^ are often used to quantify the contributions of each physical variable and elementary reaction to CEM, thereby indicating the key independent variables and reactions that govern the ignition process.

1 $$\mathrm{EI}=\frac{\left|diag\left(a_{e}b_{e}\right)\right|}{\sum \left|diag\left(a_{e}b_{e}\right)\right|}$$

2 $$\mathrm{PI}=\frac{\left|\left(b_{e}S_{t}\right)⊗R\right|}{\sum \left|\left(b_{e}S_{t}\right)⊗R\right|}$$

where diag(·) returns the diagonal elements of the input matrix. ***S***_**t**_ represents the stoichiometric coefficient matrix, ***R*** represents the net reaction rate vector, and “⊗” denotes the elementwise multiplication between binary vectors.

Furthermore, CEMA enables the rigorous identification of local combustion modes (LCM) resulting from chemical/diffusion interactions in the CEMA theory framework. Xu et al.^41^ proposed an indicator α = ϕ_s_/ϕ_ω_, which discerns the local combustion mode by examining the projected contribution of molecular diffusion in the eigenvector direction associated with the CEM, as formulated below:

3 $$\alpha =\phi_{s}/\phi_{\omega },,\phi_{s}=b_{e}\cdot s,,\phi_{\omega }=b_{e}\cdot \omega$$

where ***s*** and **ω** denote the diffusion and chemical source terms, respectively. The mixture with |α| < 1 and ignorable importance of diffusion versus chemistry is defined as the autoignition mode (AUTO). The mixture satisfying α > 1 with the diffusion dominating over chemistry is defined as diffusion-assisted ignition (DIFF), and the one satisfying α < −1 with diffusion dominating but unfavorable for ignition chemistry is referred to as the local extinction mode (EXTC), indicating diffusion opposing ignition chemistry.

Furthermore, the diffusion index (DI), as formulated in eq 4, can be used to identify the governing diffusive subprocesses for the local combustion mode:^42^

4 $$\mathrm{DI}=\frac{b_{e}⊗s}{\max_{1\leq i\leq K+1}\left|b_{e}⊗s\right|}$$

DI varies within [−1, 1], and a larger value of DI absolute indicates that the species diffusion (1 ≤ *i* ≤ *K*, where *K* denotes the total number of species) or heat conduction (*i* = *K* + 1) is significant for the local ignition chemistry.

## 4 Results and Discussion

### 4.1 Dependence of Ignition Delay on Variable Stratifications

It is well recognized that the employment of cylinder charge stratification and turbulence intensity is effective to regulate the engine ignition timing,^16,18–20,41^ which is critical for overall engine performance. The ignition timing depends not only on the detailed chemical kinetics but also on the diverse combustion events or modes existing inside the cylinder, such as spontaneous ignition and deflagrative front propagation, local extinction/reignition, and detonation interaction, etc. In this section, the ignition timing behavior with various turbulence stratifications is first elucidated by chemical kinetics analysis, followed by the detection of the underlying physics responsible for the multimode phenomena in ignition.

The evolutions of spatially averaged temperature and heat release rate (HRR) in the 2D stratified turbulence domain are compared with those occurring in the 0D homogeneous reactor to clarify the dependence on stratification inhomogeneity. Here, the 0D homogeneous ignition was simulated using the SENKIN code^43^ with the initial parameters kept the same as the nominal initial means of the 2D-DNS counterpart. The critical ignition point is defined as the location of the maximal HRR in 0D homogeneous ignition or the maximal HRR_mean_ in 2D stratified ignition (HRR_mean_ is the spatial mean of HRR). Figure 3a shows the comparison of the 0D and 2D ignition delay times, and Figure 3b shows the maximal HRR and HRR_mean_ at the ignition moment, for cases 1–10. It is clearly shown in Figure 3a that for cases 1–8 with small normalized temperature fluctuations (*T*′/⟨*T*⟩ = 0.0278), stratified ignition occurred later than that in the homogeneous case. However, in cases 9/10 with intermediate or large normalized temperature fluctuations (*T*′/⟨*T*⟩ = 0.0556/0.0926), the stratified mixture ignition was dramatically advanced. In our previous publication,^20^ the stratified ignition mode was primarily determined by the temperature fluctuation intensity. At low *T*′ conditions, the spontaneous ignition mode was dominant, such that temperature or composition inhomogeneity led to an asynchronous ignition sequence, and thus, the global ignition time was delayed. However, at intermediate or high *T*′ conditions, the deflagration propagation mode was dominant, i.e., the incipient flame kernels with peak initial temperatures were first formed by the spontaneous reactions because of the leading sensitivity of ignition timing to the initial temperature, and then the incipient flame kernels propagated quickly as a deflagration front toward the neighboring low-temperature mixtures before its spontaneous reactions commenced. As a result, the stratified ignition timing was delayed in cases 9/10 (*T*′ = 60 K/100 K, respectively). It is worth noting that the deflagration mode and ignition advancement are not present in cases 7/8 with large equivalence ratio fluctuations (ϕ′ = 0.2/0.3) because of the insensitivity of ignition timing with respect to the equivalence ratio variation. However, Figure 3b depicts that the HRR peak was dramatically mitigated with either temperature or composition stratification, demonstrating the effectiveness of mixture stratification in mitigating the pressure rise rate inside the HCCI engine cylinder, especially at high *T*′ conditions (say case 10) due to the moderate temperature rise characteristic of deflagrative waves.

**Figure 3:**
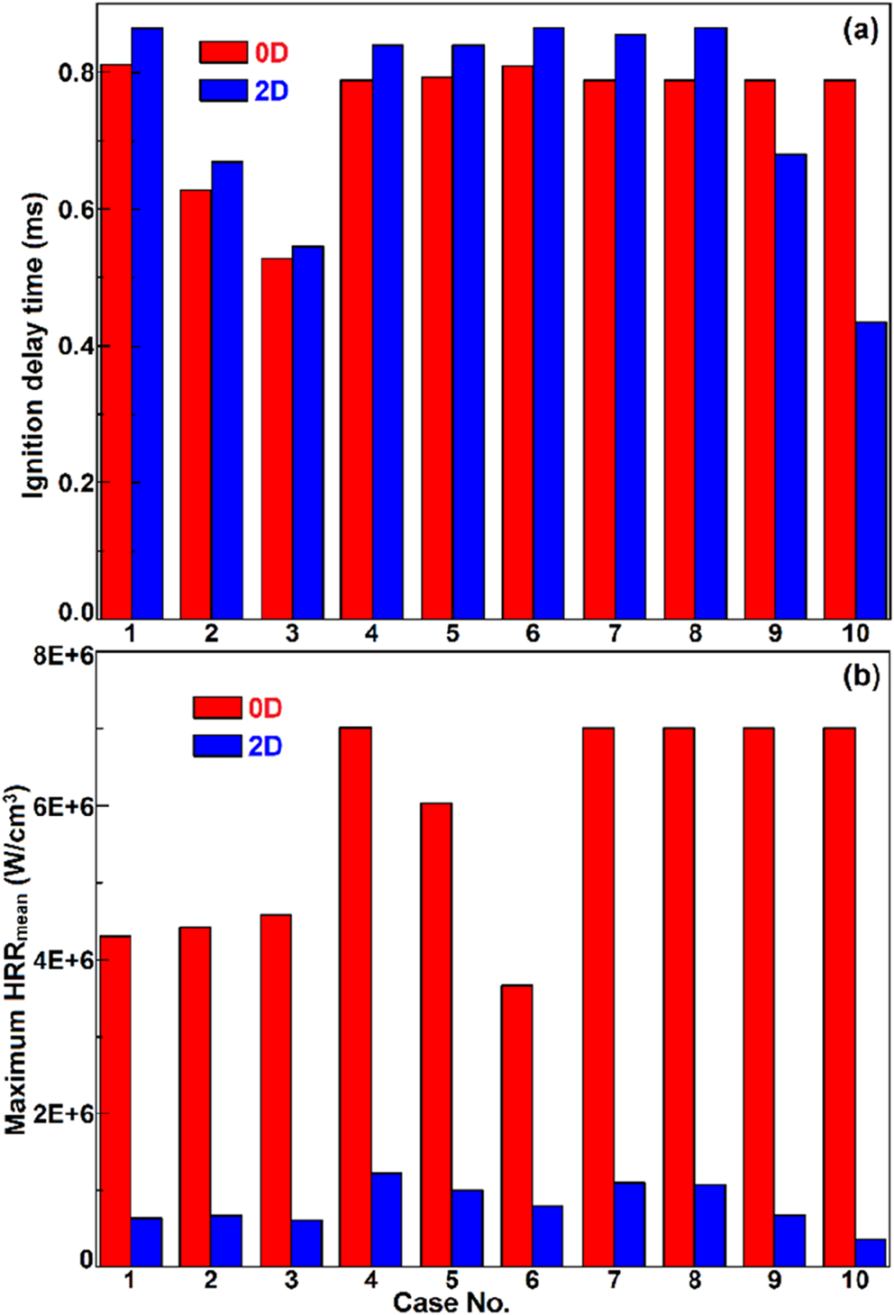
Comparisons of the 0D and 2D ignition delay times (a) and maximum HRR_mean_ (b), among cases 1–10. It is noted that the 0D homogeneous ignition data of cases 7–8 with varying ϕ′ and cases 9–10 with varying *T*′ are absolutely identical to those of case 4.

To investigate the chemical kinetics subject to the impacts of flame propagation and turbulent mixing in case 10, we draw the reaction pathway network, as shown in Figure 4. It is noteworthy that the chemical rates were integrated spatially across the domain and then temporally over the whole ignition period; therefore, they already contained the impacts of diffusive mixing and multistage evolution and differed from the pure reaction term. The arrows in different colors denote pathways in different temperature ranges (red for *T* > 1500 K, blue for *T* < 1500 K, and black for either). It is also noteworthy that the pathways commencing at NH_3_ and H_2_ designate distinct element flux routes from the fuel. It is clearly shown in Figure 4 that ignition in the low- and high-temperature ranges proceeds via distinct pathways involving different participating intermediates. For ammonia oxidation, it is oxidized with OH to the amino radical (NH_2_) via the dehydrogenation reaction (R164: NH_3_ + OH = NH_2_ + H_2_O), which is the most important exothermic reaction, and is used as the reference base for the normalization of the reaction rates of the remaining pathways. The subsequent oxidation pathways following the amino radicals, in the low- and high-temperature ranges, are fairly different. In the low-temperature, preignition stage, amino is oxidized through NH_2_ → N_2_H_4_ (+HO_2_) → N_2_H_3_ (+O_2_) → N_2_H_2_ and NH_2_ (+HO_2_) → H_2_NO (+HO_2_/NH_2_) → HNO (+NH_2_) → NO, with the former pathway predominating since the rate-controlling reaction (R97: 2NH_2_(+M)=N_2_H_4_(+M)) is a pressure-dependent third-body falloff reaction that has a fairly small activation energy at elevated pressure conditions. The global reactions from NH_3_ to NH_2_ and the subsequent pathways can be written as 2NH_3_ + 3OH + 3HO_2_ = 5H_2_O + H_2_O_2_ + 2NO, which is chain termination. As a result, the HRR intensity in the preignition stage is rather weak.

**Figure 4:**
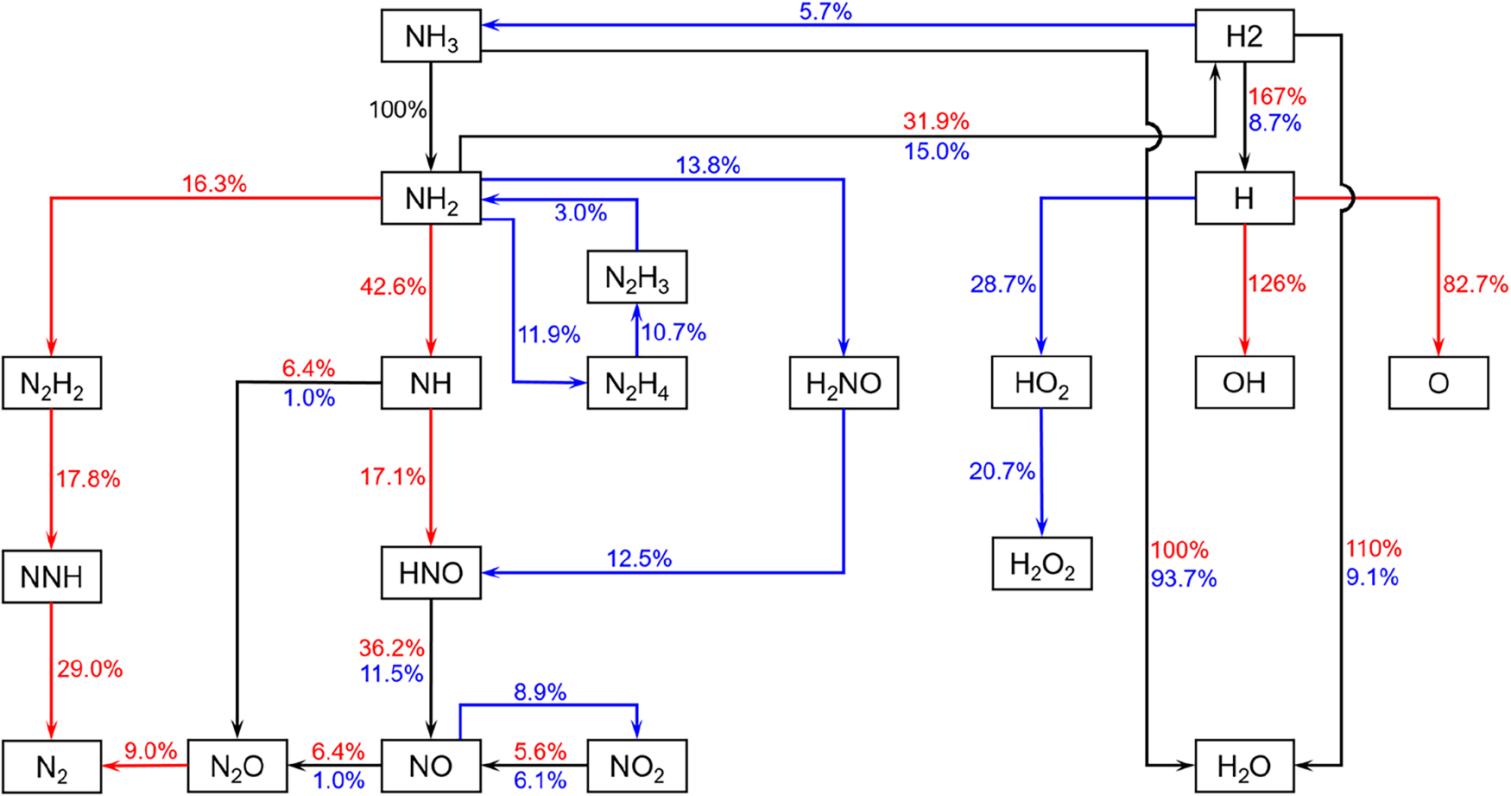
Reaction pathway network for case 10. The by-arrow percentages represent the spatiotemporal chemical rates normalized by the ammonia decomposition rate via NH_3_ → NH_2_, with a cutoff threshold of 5%. The red and blue arrows denote high-temperature (*T* > 1500 K) and low-temperature (*T* < 1500 K) pathways, respectively, and black arrows denote the common pathways.

Under high-temperature conditions after ignition, NH_2_ is oxidized through two dehydrogenation pathways, i.e., NH_2_ (+OH/H) → NH (+OH/H_2_O) → HNO (+M/OH/H) → NO and NH_2_ → N_2_H_2_ (+M) → NNH (+M) → N_2_, with the NH pathway being dominant, and hence, impeding the amino low-temperature chemistry. It is evident that HO_2_ is the most prevailing radical in the preignition stage, and OH/H becomes predominant in the postignition stage, which arises from the competition of branching chemistry involving H_2_ oxidation. More specifically, at either low- or high-temperature conditions, H_2_ in the fuel stream is always oxidized by the dehydrogenation reaction R13: H_2_ + OH = H_2_O + H; however, the H radical proceeds via different pathways in the subsequent oxidation processes. R9 (H + O_2_ = OH + O) with a pretty high activation energy is dominant at high-temperature conditions, while the third-body reaction R10 (H + O_2_ (+M) = HO_2_ (+M)) with a zero energy barrier becomes dominant at low-temperature conditions. As a result, HO_2_ and H_2_O_2_ are accumulated in the preignition and low-temperature stages, and interplay as the prevailing oxidizing radicals. Although H_2_O_2_ can decompose to OH via R22 (H_2_O_2_ (+M) = 2OH (+M)), most of the OH radicals are quickly consumed by NH_3_, thereby resulting in a deficiency of OH radicals as needed to activate the high-temperature reactions. However, in the postignition stage, R9 becomes more competing with R10 for OH production and thus triggers the high-temperature reactions; simultaneously, HO_2_ as well as its participating pathways are diminished. Besides, it is also noteworthy that the chemical rates in H_2_-involving pathways at high-temperature conditions exceeded those of the NH_3_ → NH_2_ pathway, while those at low-temperature conditions are rather decreased. Hence, H_2_ chemistry primarily commences at the high-temperature stage.

At high-temperature conditions, a notable portion of the product NO is reduced to N_2_ via the reburning pathway, i.e., R106 (NH + NO = N_2_O + H) and then R105 (N_2_O + H = N_2_ + OH). However, the NO reburning chemistry at low-temperature conditions is fairly negligible, which differs from that of ref 44, reporting a remarkable concentration of N_2_O in the product of a laminar stretched NH_3_/H_2_/air premixed weak flame that was self-sustained on the counterflow burner. This discrepancy may arise from differences in the flame configuration and parameters such as the initial equivalence ratio. Nevertheless, there is a discernible amount of NO_2_ in the low-temperature product gas, which is generated via R124 (HO_2_ + NO = NO_2_ + OH). However, under high-temperature conditions, NO_2_ is indiscernible because the reactant HO_2_ becomes fairly diminished. Additionally, at low-temperature conditions, the hydrogenation reaction of NH_2_ via R71 (NH_2_ + H_2_=NH_3_ + H) produces NH_3_, indicating that ammonia oxidation is inhibited by H_2_ addition. However, at the high-temperature ignition stage, this reaction proceeds in the reverse direction, which is responsible for the enhancement of the H_2_ concentration at the ignition point. The jet-stirred reactor experiments operating under low-temperature (1000–1250 K) conditions^45^ also indicate a similar inhibitory effect of H_2_ on NH_3_ oxidation.

In a brief conclusion, the low and intermediate temperature combustion is controlled by the chain-branching process involving HO_2_ and OH radicals, resembling that in the warm flame of hydrocarbon fuels.^46^ However, the OH fraction is severely inhibited due to the rapid consumption by the NH_3_ dehydrogenation reaction (R164). Consequently, the reactions governing OH generation are the rate-limiting steps for the low and intermediate temperature HRR, and thus control the transition from low to high-temperature reactions. Figure 5 displays the conditional mean of the OH production rate in the temperature space for case 10, which indicates clearly the negative temperature-dependent production rate of OH via R124 and R173 with increasing temperature in the low-temperature range (*T* < 1500 K), thereby impeding the transition to high-temperature chemistry. It is also noteworthy that the chemical rates of R124 and R173 are highly dependent on the HO_2_ concentration, which is highly associated with the low-temperature reactions and secondary explosion limit of hydrogen at elevated pressures. On the contrary, OH production through the branching reactions R9, R14, and R22 exponentially grows with temperature; thus, they surpass R124/R173 and become dominant in the high-temperature range (*T* > 1500 K), eventually leading to ignition chemistry and thermal runaway explosion, although they also govern the endothermic reactions.

**Figure 5:**
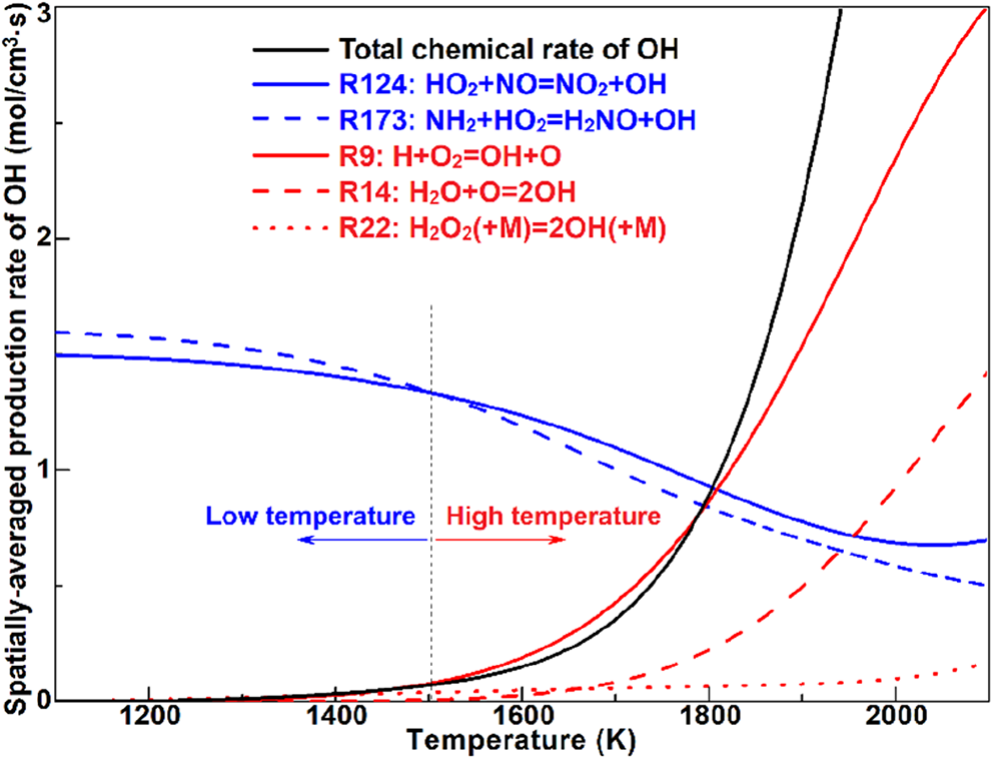
Spatially averaged production rate of OH due to different governing reactions conditioned with respect to temperature in case 10.

### 4.2 Detection of the Ignition Dynamics of the NH_3_/H_2_/Air Stratified Mixture Using CEMA

In this section, we investigate the ignition dynamics of the NH_3_/H_2_/air stratified mixture with the underlying physics using the CEMA diagnostic tool. To verify the rationality of the CEMA theoretical framework, we applied it to analyze the NH_3_/H_2_/air mixture ignition process in a homogeneous reactor at *p*_0_ = 40 atm, *T*_0_ = 1080 K, ϕ_0_ = 0.8, and ω_H_2__ = 0.1, as illustrated in Figures 6 and 7, respectively. As shown in Figure 6a, in the preignition stage, the CEM mode associated with a positive real-part eigenvalue (λ_e_), representing the explosiveness and thermal runaway feature of the unburnt mixture, exists in the reaction system, which drags the fresh mixture to ignition. As shown in Figure 6b, once ignition occurs, the positive eigenvalue disappears abruptly and all of the active modes decay with time, which drags the postignition mixture toward the equilibrium state. Therefore, the point where Re­(λ_e_) = 0 (marked by a magenta “+”) in Figure 6a,b can be used as an ideal definition for the critical ignition point, which was approved agreeing pretty well with the maximal HRR point that was widely used as the definition of ignition in the literature.^47,48^ Figure 7 depicts the evolution of the dominating variable EIs during the ignition process, where the variable dominance is defined based on its parallelization with the CEM mode. This indicates that temperature, as well as the low-temperature species, including HO_2_, H_2_O_2_, NH_2_, and H_2_NO, play a leading role in the preignition reactions, while in the postignition stage, the product NO becomes the most significant scalar. The dominant species identified by EI, as well as the dominating reactions revealed by PI agree fairly well with the previous chemical pathway analysis, demonstrating that CEMA is a systematic and rigorous tool for the detection of reaction systems.

**Figure 6:**
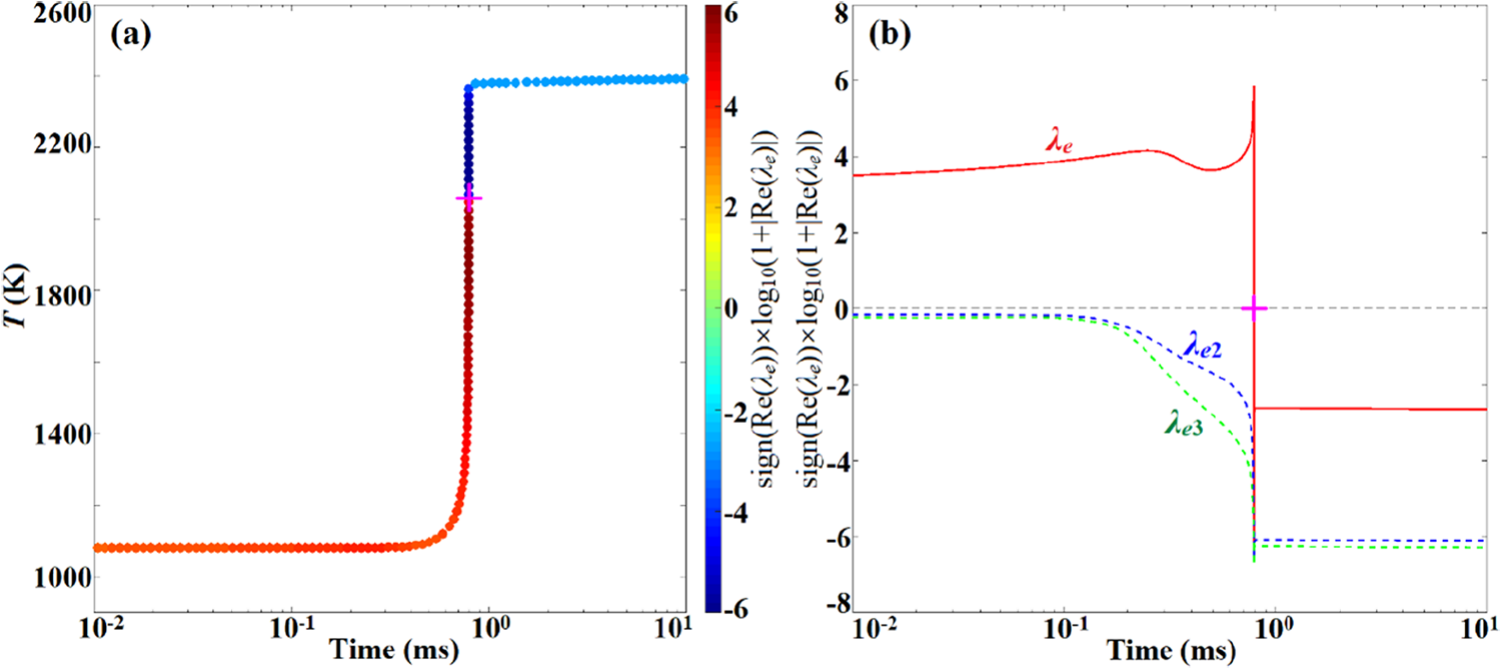
Evolutions of temperature and logarithmic real-part eigenvalues in the ignition process simulated by Senkin at *p*_0_ = 40 atm, *T*_0_ = 1080K, ϕ_0_ = 0.8, and ω_H_2__ = 0.1 (a). In (b), λ_e_ denotes the first eigenvalue with the largest real part associated with the CEM, and λ_e2_ and λ_e3_ are the second and third eigenvalues with decreasing orders of magnitude. The magenta symbol “+” denotes the critical ignition point defined by Re­(λ_e_) = 0.

**Figure 7:**
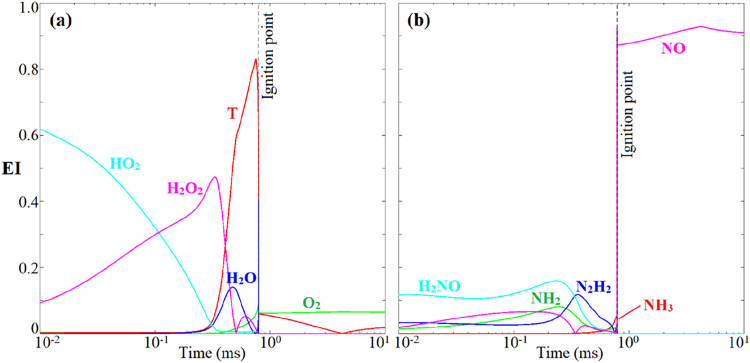
EIs of the top dominating variables ((a) for T, HO_2_, H_2_O_2_, H_2_O, O_2_, and (b) for NO, H_2_NO, NH_3_, NH_2_, N_2_H_2_), with the remaining ones with EI < 0.03 omitted. The vertical dashed line denotes the ignition point identified by Re­(λ_e_) = 0.

As previously concluded, the ignition in cases 9/10 with intermediate or high-temperature stratification was governed by the deflagration mode, while the other cases with lower temperature stratification were governed by spontaneous ignition. Here, cases 1 and 10 are selected for CEMA analysis to elucidate the physical characteristics of these two distinct ignition modes. Figure 8 shows the profiles of temperature, and logarithmic CEM eigenvalue (λ_e_) and Damköhler number (*Da*), at different progress variables (*c̅*) defined based on the temperature rise (i.e., *c̅* = (*T*_mean_ – *T*_0_)/(*T*_equi_ – *T*_0_), where *T*_mean_ is the spatial mean of temperature, *T*_equi_ is the equilibrium temperature, and *T*_0_ = 1080 K is the initial temperature). In each subfigure, the first progress variable corresponds to a moment with the formation of incipient flame kernels, and the last corresponds to the ignition moment. It is also noteworthy that the *Da* number, defined as the ratio of the turbulence flow time scale to the reaction time scale (Re­(λ_e_)^−1^), as expressed in eq 5,^15^ can quantify the relative importance of turbulence mixing against the reaction and thus indicate the local ignition mode:

5 Da=χ−1/Re(λe)−1=Re(λe)·χ−1

In this article, the turbulence flow time scale is defined as the reciprocal of the scalar dissipation rate (χ), as defined in eq 6:

6 $$\chi =2D|\nabla \xi {|}^{2}$$

where *D* is the local mixture thermal diffusivity and ξ is the mixture fraction, as formulated in eq 7:

7 $$\xi =\frac{0.25\frac{Y_{H}-Y_{H,\mathrm{oxid}}}{W_{H}}+0.75\frac{Y_{N}-Y_{N,\mathrm{oxid}}}{W_{N}}-0.25\frac{Y_{O}-Y_{O,\mathrm{oxid}}}{W_{O}}}{0.25\frac{Y_{H,\mathrm{fuel}}-Y_{H,\mathrm{oxid}}}{W_{H}}+0.75\frac{Y_{N,\mathrm{fuel}}-Y_{N,\mathrm{oxid}}}{W_{N}}-0.25\frac{Y_{O,\mathrm{fuel}}-Y_{O,\mathrm{oxid}}}{W_{O}}}$$

where *Y*_*i*_ and *W*_*i*_ are the mass fraction and mole weight of element *i*, respectively, and subscripts fuel and oxid designate the fuel and oxidizer streams, respectively.^49^ The reaction time scale is defined as the reciprocal of the CEM eigenvalue (λ_e_).

**Figure 8:**
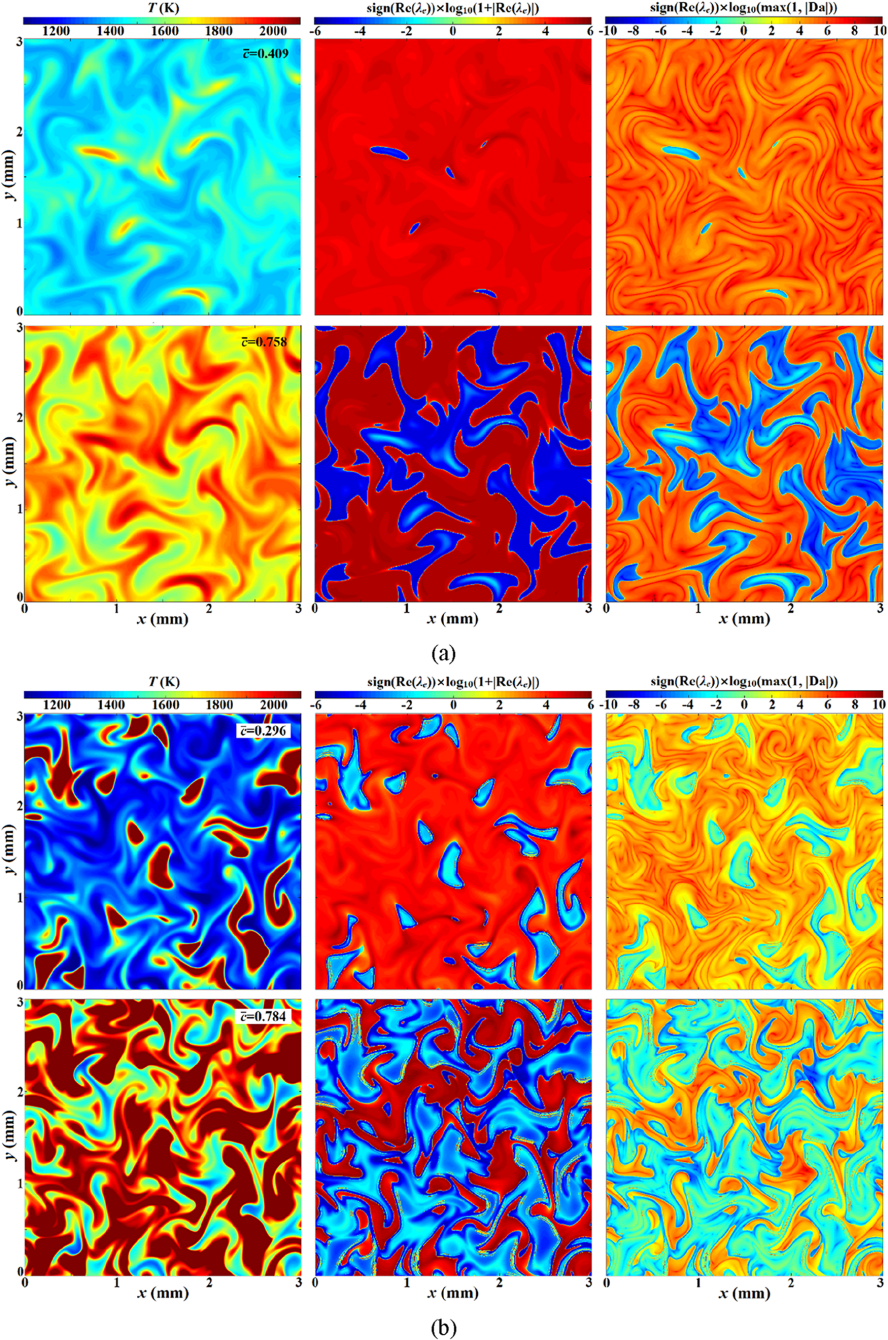
Instantaneous profiles of temperature, logarithmic eigenvalue (λ_e_), and *Da* number at different progress variables (*c̅*) for cases 1 (a) and 10 (b).

In this study, it is suggested that fluids with *Da* > 5 are governed by the spontaneous ignition mode, and those with *Da* < 5 are governed by the deflagration mode. Additionally, it was found that the resultant conclusions were fairly insensitive to the change of this threshold. It is seen in Figure 8 that the fresh, unburnt mixtures are characterized by positive eigenvalues, elucidating their chemical explosiveness nature. The hot pockets after ignition have negative eigenvalues, with the absolute eigenvalue controlling the reaction rates toward the equilibrium state. In either case, the exhaust zone engulfs the unburnt zone gradually until the ignition ends. Remarkably, much finer structures were generated in the deflagration mode (case 10) than in the spontaneous ignition mode (case 1). This may arise from the strong thermal dilation in case 1 due to spontaneous ignition, which tends to uniformize the reacting front morphology. With respect to case 10, the combustion/flame interaction is highly inhibited due to the mitigation of thermal dilation. This point can also be evidenced from the eigenvalue real-part magnitudes as shown in the second column of Figure 8, which displays that for either the pre- or postignition moment, |Re­(λ_e_)| in case 10 is always smaller than that in case 1. Namely, the preignition reactions in case 10 proceed at slower rates than those in case 1, and the system is relaxed to the equilibrium state at slower rates than those in case 1. Hence, the combustion intensity in case 10 is severely mitigated. The *Da* profiles, as depicted in the third column of Figure 8, demonstrate that in case 1, in either the pre- or postignition region, *Da* always has significant absolutes, indicating the leading role of ignition chemistry versus turbulence dissipation mixing. However, for case 10 in the deflagration mode, the *Da* values are fairly low, especially at the propagating front captured by the isocontour Re­(λ_e_) = 0,^50^ indicating that scalar dissipation competes with ignition chemistry in determining the reaction dynamics.

Figure 9 depicts the conditional statistics of the logarithmic HRR with respect to *T* at different moments in the above two distinct ignition modes. This shows that in the spontaneous ignition mode (case 1), the HRR-*T* state parameters of the reacting parcels regress onto the conditional mean curves with fairly indiscernible deviations, albeit with strong thermal dilation/flame interaction. In the deflagration mode (case 10), the early moment *c̅* = 0.001 corresponding to the spontaneous ignition is also featured with indiscernible deviations in the HRR-*T* statistics. However, once the incipient propagating fronts are formed (say *c̅* = 0.296, 0.784), significant discrepancies are observed in the high-temperature region. This is because, in the deflagration mode, the combustion regime of the fresh mixtures upstream of the leading front has been altered to diffusion-forced ignition, rather than spontaneous ignition. Consequently, it is suggested that the remarkable statistical deviations of the state parameters arise from diffusion, which dominates the ignition chemistry.

**Figure 9:**
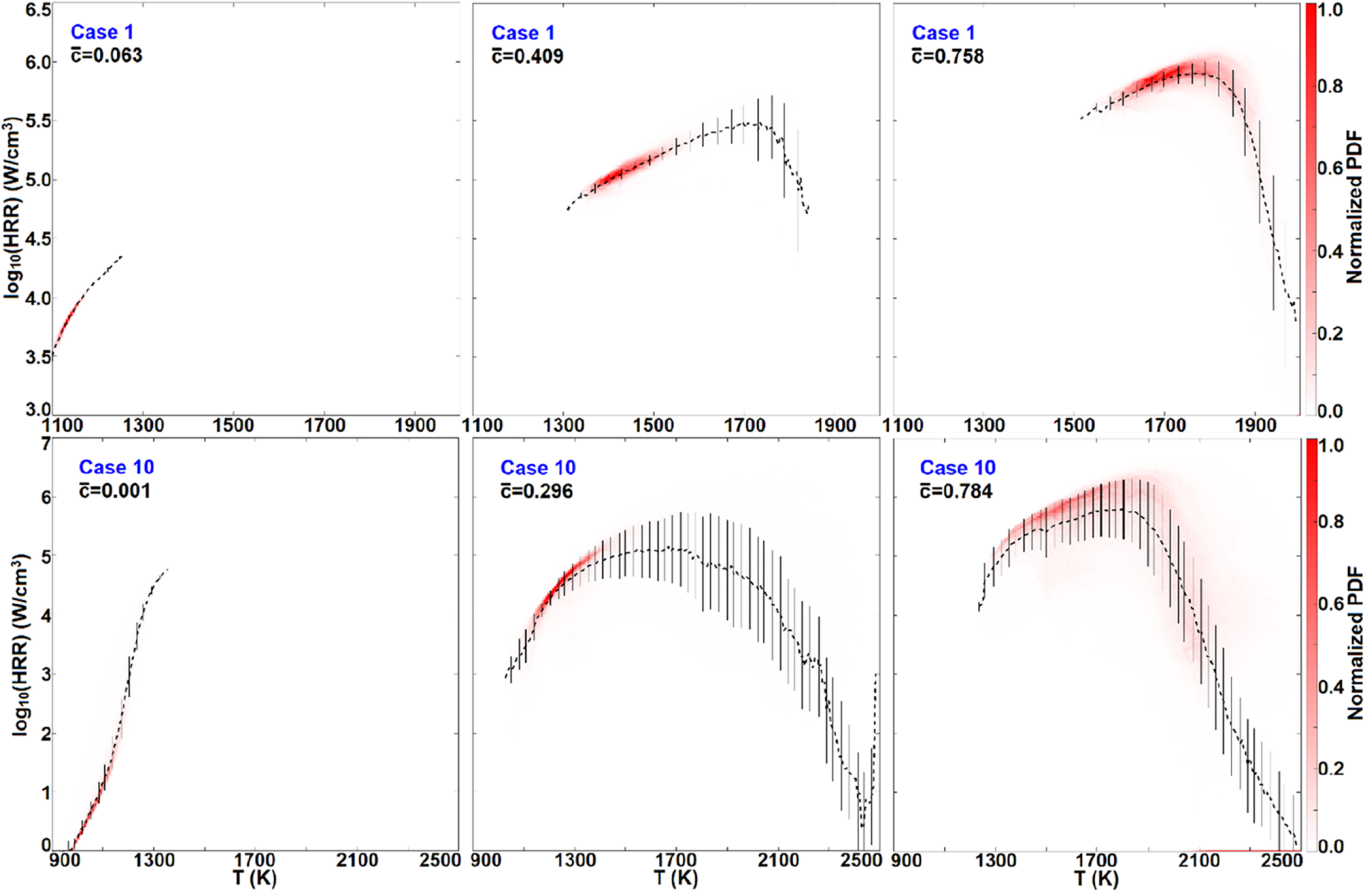
Conditional statistics of the logarithmic HRR with respect to *T* at different instants in cases 1 and 10, respectively, with the nonreacting parcels satisfying ξ < 10^–3^ omitted. The color indicates the normalized joint PDF magnitude.

### 4.3 Detection of the Ignition Modes Using the CEMA

This section emphasizes the fundamental physics underlying the ignition modes that exist in turbulent stratified NH_3_/H_2_/air mixture combustion by examining the local combustion mode (LCM) distributions using CEMA. To aid interpretation, a laminar premixed flame is first analyzed, which serves as a reference for comparison with the DNS databases. Figure 10 illustrates the distributions of LCMs inside the premixed flame front, with the incoming flow parameters equaling the nominal initial means of case 10, i.e., *p*_0_ = 40 atm, *T*_0_ = 1080 K, ω_H_2__ = 0.1, and ϕ_0_ = 0.8. With the elevated inlet temperature, the flame burning velocity is not yet a constant but becomes highly dependent on the inlet induction length,^42^ which refers to the distance from the inlet boundary to the propagating front. Spontaneous reactions occurring within the upstream induction domain control the flame stabilization and propagation; hence, the premixed flame in this circumstance is governed by the autoignition mode. As shown in Figure 10a, in the leading front with the existence of CEM, i.e., the sheet region just ahead of the front, where Re­(λ_e_) = 0 marks the ignition threshold, the projected contribution of ignition chemistry (ϕ_ω_) is significantly higher than that of diffusion (ϕ_s_), implying that the flame propagation speed is governed by the spontaneous reactions. In the PREMIX code^51^ simulation, if the inlet induction length was set to the turbulence integral scale (*l*_t_ = 0.4 mm), the resultant spontaneous ignition wave speed was 105.1 cm/s, which is comparatively faster than the deflagration propagation speed, which is present at a lower inlet temperature and is independent of the inlet domain length. Figure 10b shows the profiles of some typical reactive scalars inside the premixed flame sheet, governed by spontaneous reactions.

**Figure 10:**
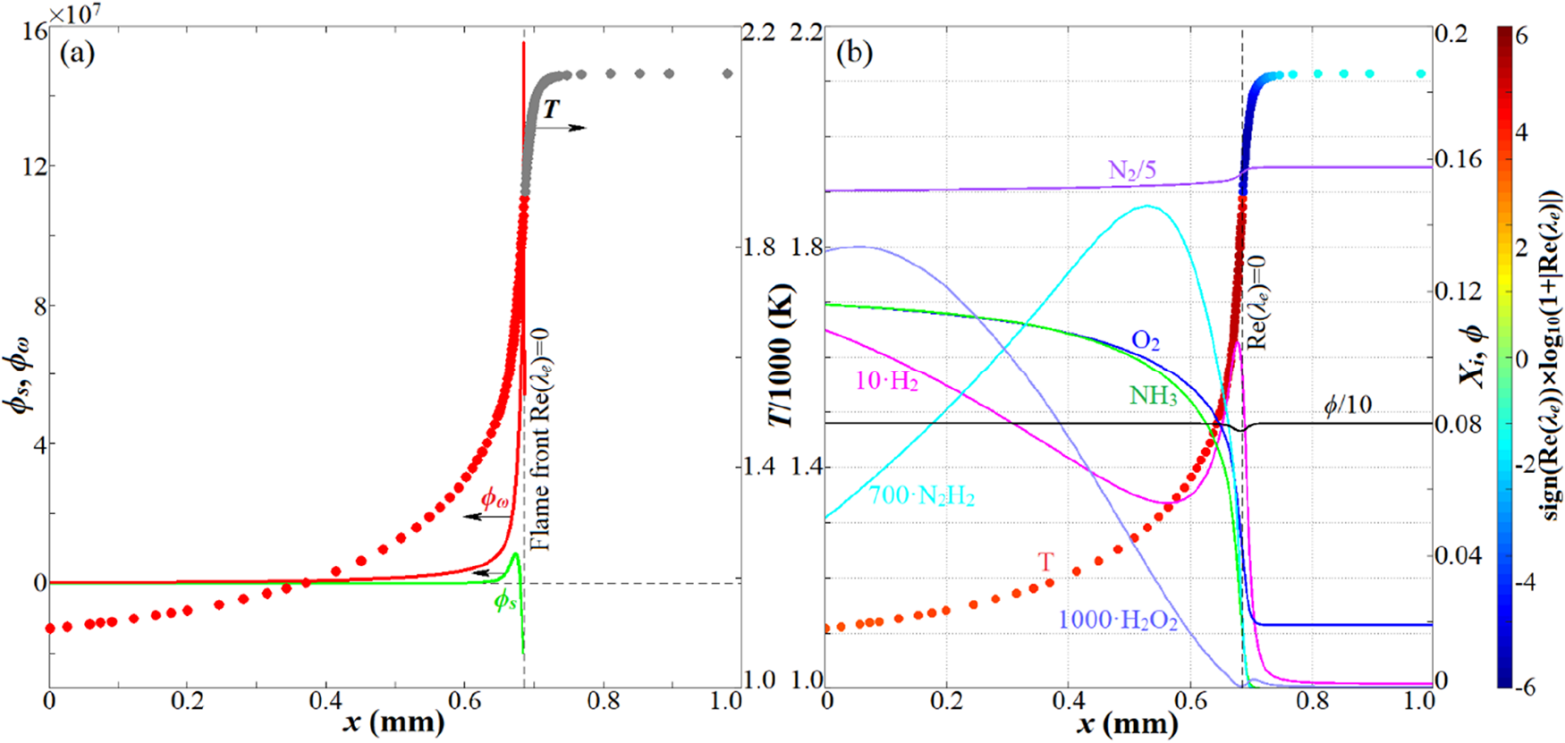
Distributions of LCM (a) and reactive scalars (b) inside the NH_3_/H_2_/air laminar premixed front in the autoignition mode. *p*_0_ = 40 atm, *T*_0_ = 1080 K, ω_H_2__ = 0.1, and ϕ_0_ = 0.8.

Figure 11 displays the instantaneous distributions of LCMs identified by the local combustion indicator α defined by formula [Disp-formula eq3], in cases 1 and 10. For each case, the first progress variable *c̅* corresponds to the moment of formation of the incipient flame kernels, and the second corresponds to the ignition moment with maximal HRR_mean_. The gray color indicates the postignition (POST) zone with Re­(λ_e_) < 0, and the preignition zone with Re­(λ_e_) > 0 is separated into the autoignition zone (AUTO) in red, the diffusion-assisted ignition zone (DIFF) in green, and the local extinction zone (EXTC) in blue. It is worth noting that the border of the POST zone satisfying Re­(λ_e_) = 0 can be regarded as the propagating front. It can be seen that in the whole ignition period of case 1, only the AUTO mode was dominant in the leading front with Re­(λ_e_) > 0, indicating that the effectiveness of thermal/mass diffusions on flame propagation was unimportant, even in the propagation front region. Hence, case 1 was governed by the spontaneous ignition mode. As a verification, we performed an NH_3_-based transport budget analysis along cutline C that is sampled in case 1 (as depicted in Figure 11), and the results are shown in Figure 12a, which indicates the dominance of chemical rates versus diffusion and thus the governance of the spontaneous ignition mode. In case 10, it can be seen that the DIFF and EXTC modes emerged at the leading side of the propagation front, with the DIFF mode ubiquitously existing, which proves that diffusion across the reacting front promoted local ignition and flame propagation. The transport budget analysis along cutline B in case 10, as shown in Figure 12b, depicts comparative magnitudes of diffusion with the reaction rates, which indicates the governance of the deflagration mode. Therefore, the local combustion modes can be accurately determined by the CEMA. The occasional survival of the EXTC mode was unfavorable to the local ignition chemistry, but its impact was unimportant. At the high-temperature ignition moment (*c̅* = 0.784), the diffusion effects due to the DIFF and EXTC modes diminished, with the AUTO mode playing a governing role in the ignition of the unburnt mixture, suggesting that deflagrative propagation in the early stage supported the later spontaneous ignition. More importantly, the deflagrative propagation mechanism in case 10 is dramatically different from the previous understanding. It is shown in Figure 11 that the deflagration front in case 10 consists of the “AUTO-DIFF-POST” or “AUTO-DIFF-EXTC-POST” structure from the unburnt to burnt side, which differs from the “AUTO-POST” structure in the spontaneous ignition wave in a laminar (Figure 10a) or stratified turbulence configuration (case 1). It also differs from the “DIFF-AUTO-POST” structure of the typical deflagrative propagation wave existing in laminar premixed flames.^42^ In a brief summary, the deflagrative propagation mechanism due to high-temperature stratification differs from the spontaneous ignition mode or the traditional deflagration mode in laminar premixed flames. We define this unique mechanism as the “diffusion-assisted spontaneous ignition mode”, in which diffusion ahead of the reacting front promotes spontaneous ignition and flame propagation.

**Figure 11:**
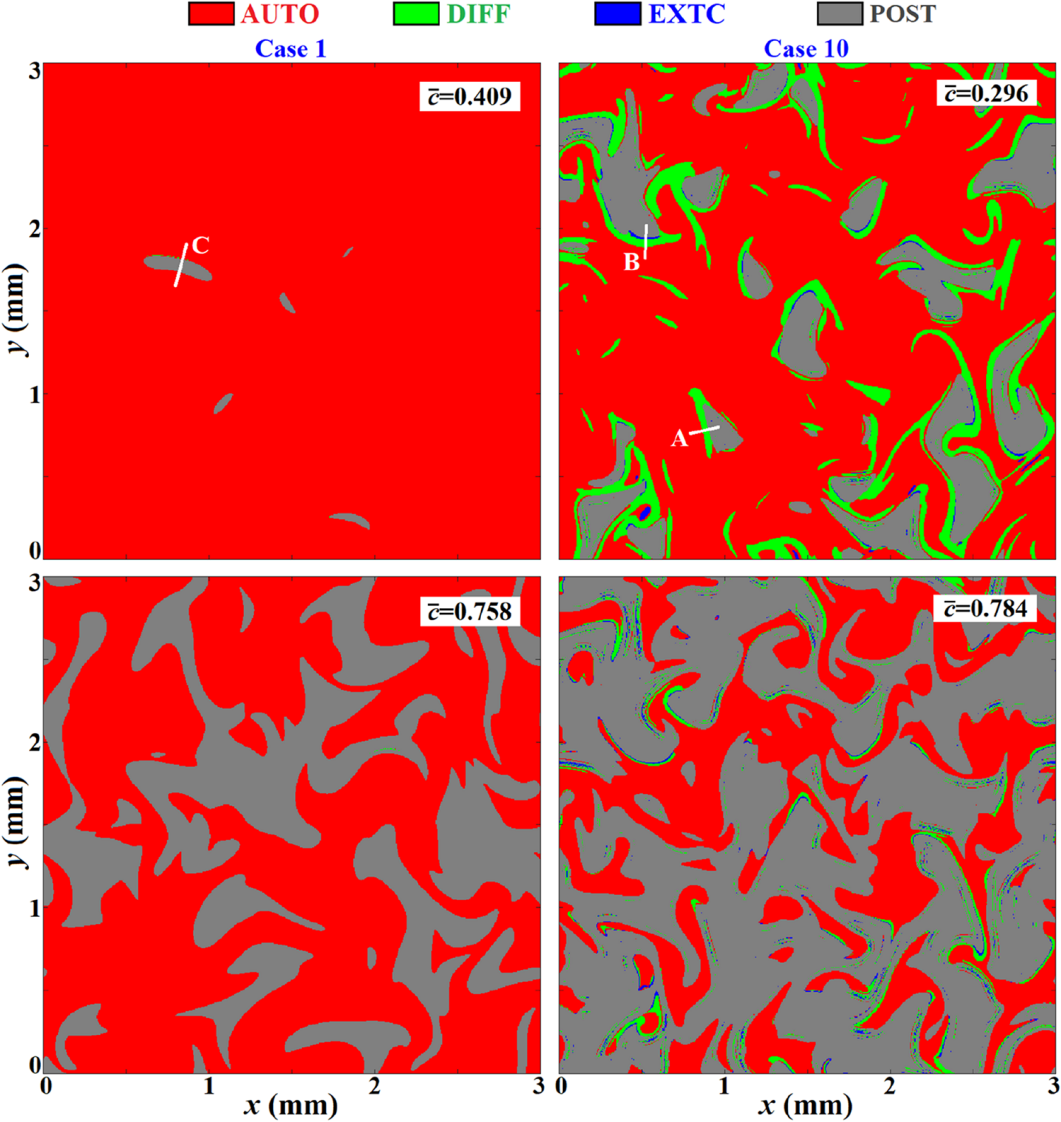
Instantaneous distributions of LCMs in cases 1 and 10. The border of the gray zones denotes the isoline Re­(λ_e_) = 0.

**Figure 12:**
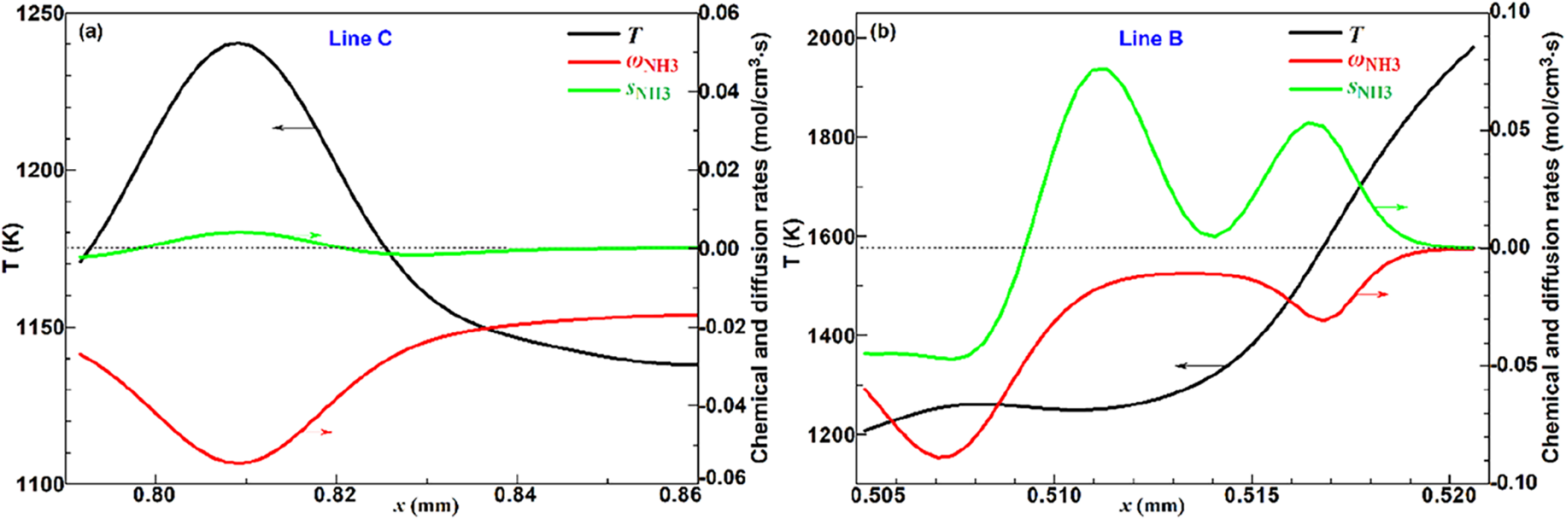
Transport budget analyses along cutlines C (a) and B (b).

Figure 13 shows the conditional PDF of the LCMs in ϕ space at some typical moments for case 10. Here, the local equivalence ratio (ϕ) is estimated on the basis of the local mixture fraction, i.e., ϕ = [ξ/(1 – ξ)]/[ξ_st_/(1 – ξ_st_)], where ξ_st_ is the stoichiometric mixture fraction. It is clearly shown that the AUTO mode remained basically motionless in the stoichiometry space, anchoring at a fuel-lean position (ϕ/(1 + ϕ) ≈ 0.42) in the overall ignition period. However, the DIFF mode formed in the low-temperature, leaner fluids moved toward richer zones within the early interval *c̅* = 0.079–0.151, demonstrating that the incipient deflagrative wave had a prominent propensity for propagation in the stoichiometry space. Figure 14 depicts the evolution of the HRR due to each LCM in case 10 ignition, and the built-in pie indicates the volume-integrated heat production rate (∮HRRd*V*). The AUTO mode plays the governing role in heat production, followed by the POST and DIFF modes in decreasing order of importance. The contribution of the EXTC mode is fairly indiscernible. The DIFF mode, which survives only in the preignition stage, promotes spontaneous reactions and flame propagation with leading significance, albeit with only a 5.47% contribution to the integrated heat production.

**Figure 13:**
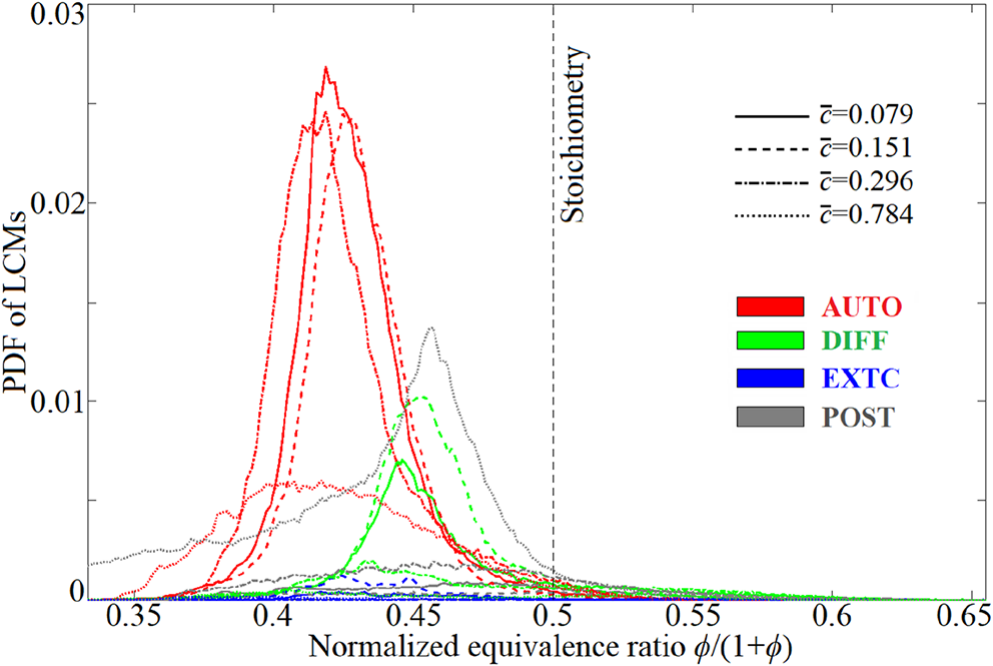
Conditional PDF of LCMs in the ϕ space at different progress variables *c̅* = 0.079 (*t* = 0.26 ms), 0.151 (0.32 ms), 0.296 (0.38 ms), and 0.784 (0.435 ms) in case 10.

**Figure 14:**
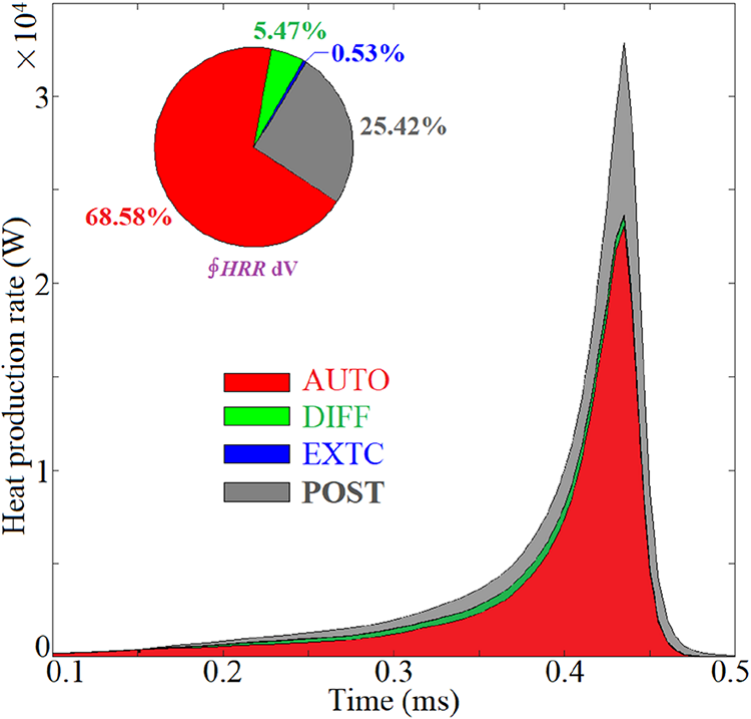
Evolution of the HRR due to each LCM in case 10 ignition.

### 4.4 Physical Mechanics Underlying the Deflagrative Front Propagation in Stratified Turbulence

Given the leading importance of the deflagration mode in regulating the stratified mixture ignition timing as well as the diversity of local modes and structures in cases involving deflagration waves, it is fundamentally and practically meaningful to investigate the physical fluid mechanics that govern deflagrative propagation in the stratified turbulence field.

In this section, we select cutlines A and B, as shown in Figure 11, as samples, which cross over the deflagration fronts, to clarify the underlying physical mechanics. It is noteworthy that cutline A consists of the “AUTO-DIFF-POST” structure, and B the “AUTO-DIFF-EXTC-POST” structure. Figure 15 displays the distributions of the dominant reactive scalars as well as their corresponding DIs along cutlines A and B. It can be seen that for case 10 with the mean equivalence ratio ϕ_0_ = 0.8, the local ϕ at the propagating front was maximal, reaching 1.12 for cutline A and 1.31 for cutline B. The local enrichment of ϕ at the propagating front arises from 2-fold aspects. First, H_2_/H was generated via the ammonia dehydrogenation sequence (R71: NH_3_ + H = NH_2_ + H_2_, R62: NH_2_ + H = NH + H_2_, R67: 2NH_2_ = N_2_H_2_ + H_2_) and R13: H_2_ + OH = H_2_O + H, respectively, at the propagating front, while being destructed elsewhere. Second, the diffusion sources of H_2_/H were also positive at the propagating front, but negative elsewhere. Hence, the preferential diffusion effect associated with H_2_/H radicals is responsible for the local enrichment of ϕ at the propagating front. More importantly, H_2_/H diffusive fluxes from the tailing-rich front to the leading leaner zone forced the upstream ignition reactions. This is the underlying physics for the unique “diffusion-assisted spontaneous ignition mode” surviving in the stratified mixture combustion, which resembles the “antisupport” effect^11,52^ that makes the instantaneous flame velocity in stratified mixtures deviate from that in the homogeneous mixtures. The DI profiles ahead of the ignition front indicate that heat conduction is the most significant for the DIFF mode, followed by NH_3_, O_2_, H_2_, and N_2_H_2_ diffusions in decreasing order of importance.

**Figure 15:**
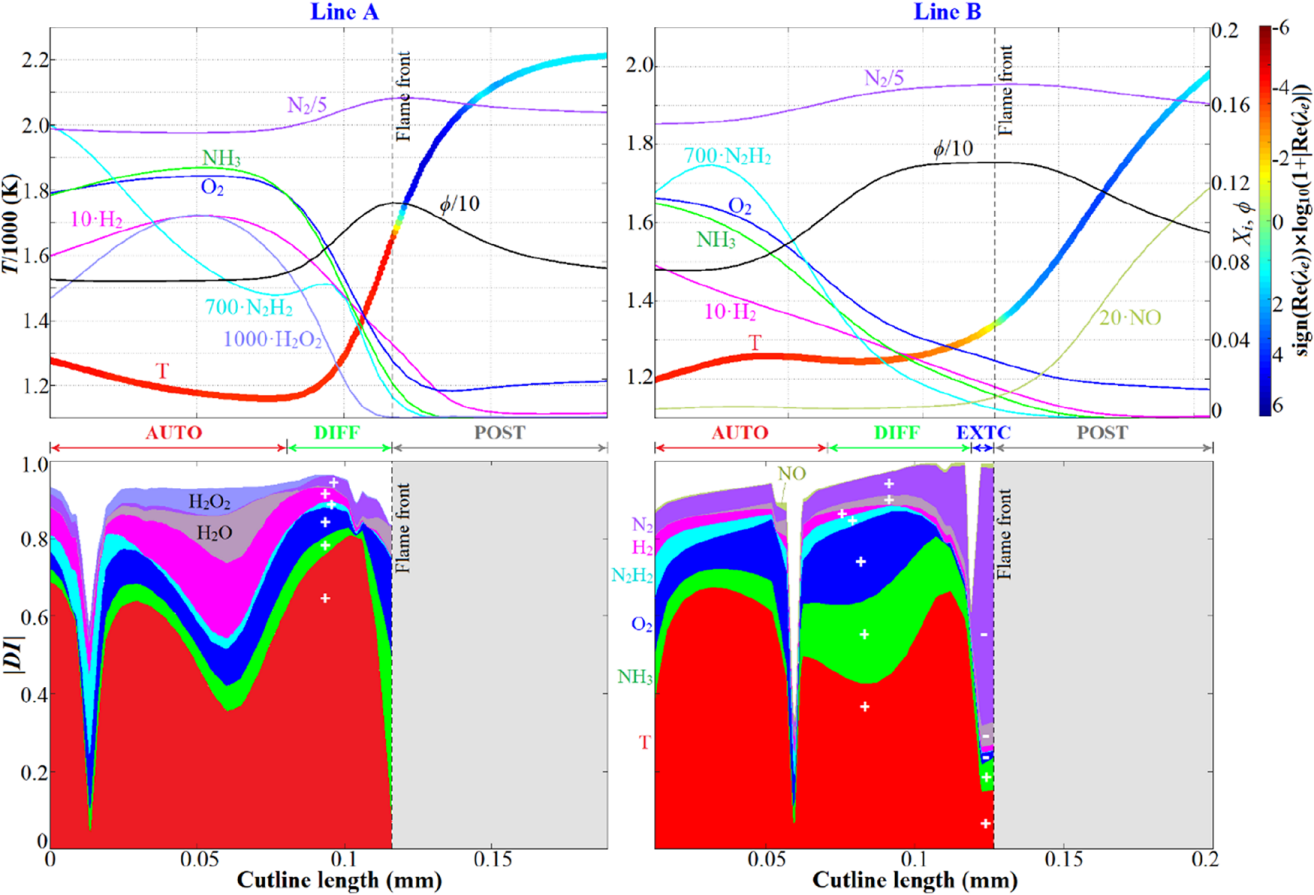
Distributions of the dominating reactive scalars (first row) with their corresponding DIs (second row) along the cutlines A and B, which cross over the deflagration fronts of case 10 (shown in Figure 10), with DIs < 0.03 omitted. The vertical dashed line denotes the propagating front identified by Re­(λ_e_) = 0. The symbols “+ and “–” indicate the sign of the DIs.

Figure 16a displays the displacement speed (*S*_d_) defined based on the *Y*_H_2_O_ iso-surface along cutline A, with *S*_d_ formulated in eq 8, where ω_H_2_O_, *W*_H_2_O_, *D*_H_2_O_, and *Y*_H_2_O_ represent the chemical rate, mole weight, diffusivity, and mass fraction of H_2_O, respectively. *n* is the unit vector normal to the flame front surface identified based on λ_e_, which points to the unburnt mixture side according to eq 8. As shown in eq 8, *S*_d_ consists of *S*_d,ω_ due to reaction, *S*_d,N_ due to normal diffusion (diffusion parallel to the normal vector *n*), and *S*_d,T_ due to tangential diffusion (diffusion perpendicular to *n*). Figure 16b displays the statistical mean of *Y*_H_2_O_-*S*_d_ of the propagating front conditioned on the local front curvature (∇·*n*).

8 $$S_{d}=\underset{S_{d,\omega }}{\frac{\omega_{H_{2}O}W_{H_{2}O}\;}{\rho \left|\nabla Y_{H_{2}O}\right|}}+\underset{S_{d,N}}{\frac{\frac{\partial }{\partial n}\left(\rho D_{H_{2}O}\frac{\partial Y_{H_{2}O}}{\partial n}\right)}{\rho \left|\nabla Y_{H_{2}O}\right|}}+\underset{S_{d,T}}{D_{H_{2}O}\left(\nabla \cdot n\right)},,n=\frac{\nabla \lambda_{e}}{\left|\nabla \lambda_{e}\right|}$$

**Figure 16:**
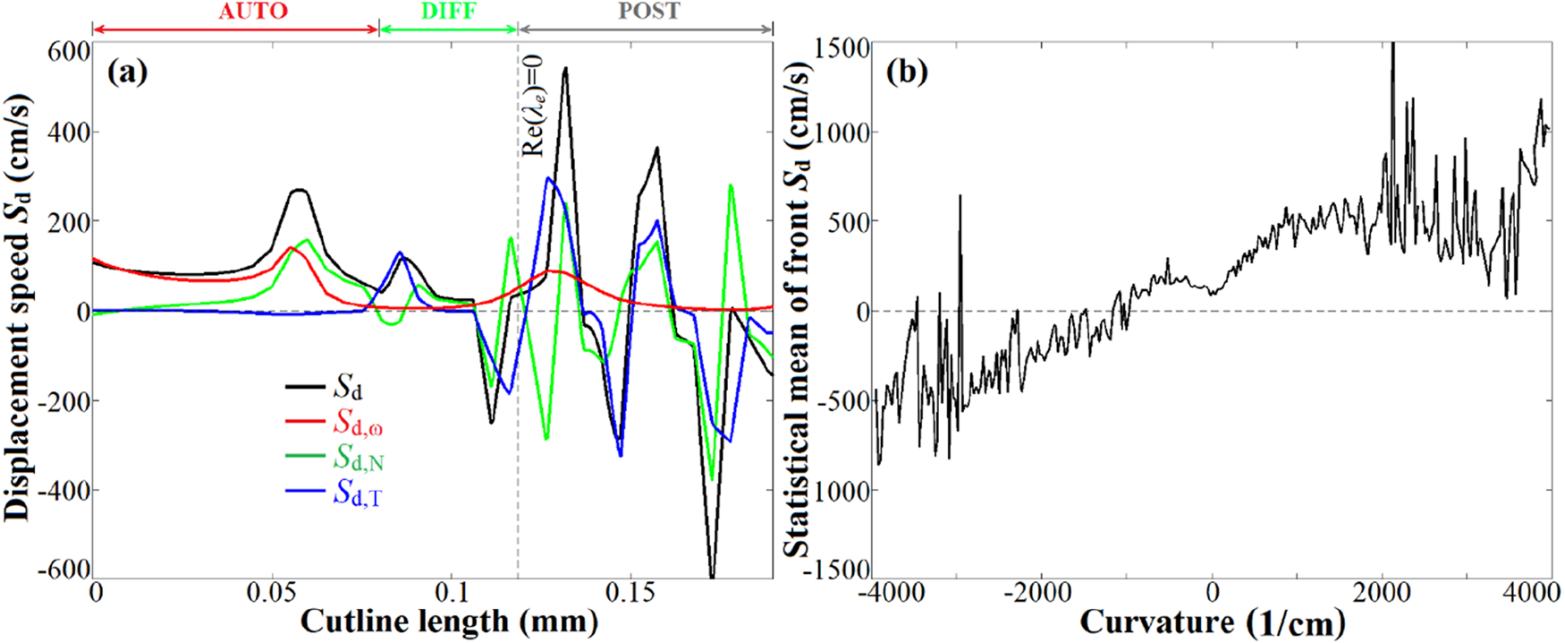
(a) *S*_d_ of the *Y*_H_2_O_ iso-surface along cutline A; and (b) statistical mean of *Y*_H_2_O_-*S*_d_ of the propagating front in the space of curvature.

Figure 16a demonstrates that the normal and tangential diffusions govern role flame propagation in the DIFF region, while the reaction and normal diffusion dominate in the AUTO region. Figure 16b exhibits a remarkable positive correlation between *S*_d_ of the propagating front and curvature, implying the leading role of curvature in accelerating the flame front propagation velocity.

To elucidate the role of reaction/diffusion in the deflagrative propagation mode with more physical meaning, we compare them by projecting onto the CEM eigendirection. ϕ_ω_ = ***b***_**e**_·**ω**, ϕ_SN_ = ***b***_**e**_· ***s***_**N**_, and ϕ_ST_ = ***b***_**e**_· ***s***_**T**_ denote the projected chemical source term, normal diffusion term, and tangential diffusion term, respectively, where ***b***_**e**_ is the left eigenvector associated with CEM. **ω**, ***s***_**N**_, and ***s***_**T**_ are vectors consisting of the chemical and normal/tangential diffusion terms appearing in the species and energy, respectively:

9 $$\frac{DY_{i}}{Dt}=\frac{1}{\rho }\frac{\partial }{\partial x}\left(\rho D_{i}\frac{\partial Y_{i}}{\partial x}\right)+\frac{\omega_{i}W_{i}}{\rho }=\underset{\omega }{\frac{\omega_{i}W_{i}}{\rho }}+\underset{S_{N}}{\frac{\frac{\partial }{\partial n}\left(\rho D_{i}\frac{\partial Y_{i}}{\partial n}\right)}{\rho }}+\underset{S_{T}}{\left|\nabla Y_{i}\right|D_{i}\left(\nabla \cdot n\right)},,n=\frac{\nabla \lambda_{e}}{\left|\nabla \lambda_{e}\right|}$$

10 $$\frac{DT}{Dt}=\frac{1}{\rho c_{p}}\frac{\partial }{\partial x}\left(\lambda \frac{\partial T}{\partial x}\right)-\frac{1}{\rho c_{p}}\sum \rho Y_{k}V_{k}c_{\mathrm{pk}}\frac{\partial T}{\partial x}-\frac{\sum \omega_{i}H_{i}}{\rho c_{p}}=\underset{\omega }{\frac{-\sum \omega_{i}\;H_{i}\;}{\rho c_{p}\;}}+\underset{S_{N}}{\frac{\frac{\partial }{\partial n}\left(\lambda \frac{\partial T}{\partial n}\right)}{\rho c_{p}\;}}+\underset{S_{T}}{\frac{\lambda }{\rho c_{p}\;}\left|\nabla T\right|\left(\nabla \cdot n\right)+\frac{\left|\nabla T\right|}{c_{p}}\left[n\cdot \sum \left(c_{\mathrm{pi}}\;D_{i}\;\nabla Y_{i}\;\right)\right]}$$

where D/D*t* is the material derivative.

Figure 17 depicts the distributions of ϕ_ω_, ϕ_SN_, and ϕ_ST_ along cutlines A and B, which clearly proves that the local modes ahead of the ignition front were primarily determined by flame curvature. More specifically, for cutline A with negative curvature (concave to the unburnt mixture side), ϕ_ST_ ≫ 0 with much larger magnitudes than ϕ_ω_ or ϕ_SN_ in the DIFF region indicates that the strong flame curvature with concave deformation to the unburnt side promotes the spontaneous ignition. For cutline B with positive curvature (convex to the unburnt mixture), ϕ_ST_ ≪ 0 appeared just ahead of the propagating front, which is responsible for the existence of the EXTC mode. This observation is consistent with the positive correlation of *S*_d_ with the curvature, as shown in Figure 16b. In the leading zones far from the propagating front, the effectiveness of tangential diffusion is significantly mitigated; thus, the AUTO mode is dominant.

**Figure 17:**
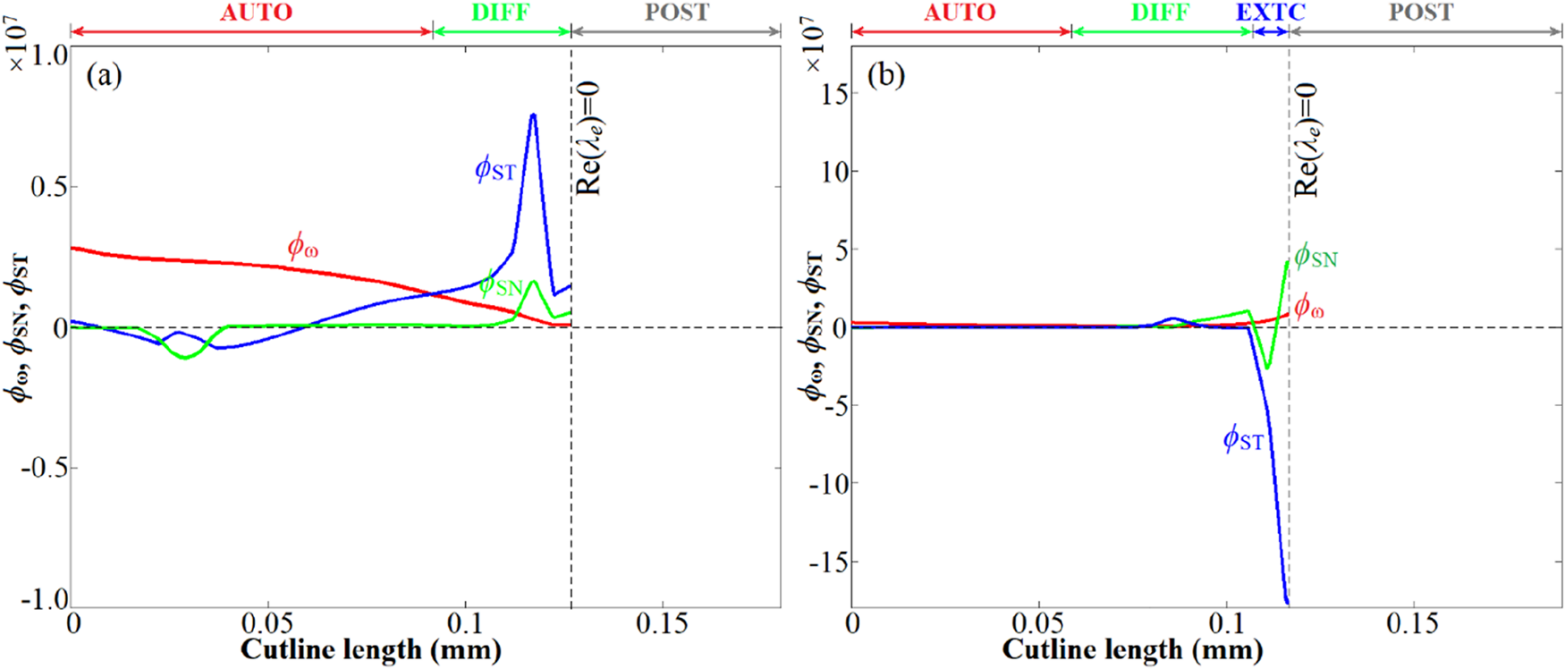
Distribution of the projected source terms ϕ_ω_, ϕ_SN_, and ϕ_ST_ along cutlines A (a) and B (b).

In summary, it is concluded that the “diffusion-assisted spontaneous ignition mode” is a unique behavior in the stratified NH_3_/H_2_/air mixture combustion with elevated temperature fluctuations, which differs from the traditional autoignition mode and deflagration mode. The preferential diffusion associated with H_2_/H radicals and tangential diffusion with a positive curvature can promote the local reaction rate ahead of the ignition front. From a practical viewpoint, we can effectively regulate the ammonia engine ignition timing by changing the hydrogen blending ratio and turbulence parameters and focusing on the impact of tangential diffusion enhanced by flame curvature.

## 5 Conclusions

In this article, CEMA is applied to the DNS databases of NH_3_/H_2_ stratified turbulent combustion to clarify the local structures and modes as well as their underlying physical mechanics. The main findings are as follows:

(1) The ignition mode of the stratified NH_3_/H_2_ mixture was primarily determined by temperature fluctuations. Low *T*′ corresponds to the spontaneous ignition mode, in which temperature or composition inhomogeneity leads to an asynchronous ignition sequence and thus the global ignition timing is delayed. Intermediate or high *T*′ corresponds to the deflagration propagation mode, which is characterized by the advancement of ignition and mitigation of the PRR.
(2) For the combustion of NH_3_/H_2_ mixtures in the spontaneous ignition mode, strong thermal dilation tends to uniformize the reacting front morphology. In the deflagration mode, the combustion/propagation front interaction is inhibited due to the mitigation of thermal dilation, thereby leaving much finer structures in the domain. Additionally, the dominance of diffusion in the deflagration mode also leads to significant statistical deviations in the state parameters.
(3) The NH_3_/H_2_ stratified combustion with high *T*′ is governed by a unique mechanism called the “diffusion-assisted spontaneous ignition mode”, in which diffusion ahead of the reacting front promotes spontaneous ignition and flame propagation. It is characterized by an “AUTO-DIFF-POST” or “AUTO-DIFF-EXTC-POST” structure that differs from the traditional autoignition mode or laminar deflagration, as well as the prominent propensity for propagation in the stoichiometry space.
(4) Preferential diffusion of H_2_/H radicals from the tailing-rich front to the leading leaner zone forced the upstream ignition reactions, which is the underlying physics for the unique “diffusion-assisted spontaneous ignition mode” surviving in the stratified NH_3_/H_2_/air mixture combustion. The local modes ahead of the ignition front are primarily determined by the flame curvature. Specifically, for the ignition fronts with negative curvature, the concave front wrinkling to the unburnt side promotes spontaneous ignition, while for those with positive curvature, the tangential diffusion with a negative contribution to ignition appears just ahead of the propagating front, thereby leading to the existence of the EXTC mode.
